# Severe pneumonia-associated lung injury: gut-lung-immune-metabolic connections in the critically ill host environment-microecology, barrier function, and mechanical ventilation

**DOI:** 10.3389/fimmu.2026.1914644

**Published:** 2026-10-21

**Authors:** Cheng Cheng, Xia ping Xiao, Lei Zhang, De Chen, Ling Lu, Ying Liu, Song Du

**Affiliations:** 1 Gansu Provincial Maternity and Child Care Hospital, Lanzhou, Gansu, China; 2 Gansu Provincial Maternity and Child Care Hospital (Gansu Provincial Central Hospital), Lanzhou, Gansu, China

**Keywords:** severe pneumonia, lung injury, gut microbiome, intestinal barrier, immune metabolism, inflammatory amplification, mechanical ventilation, ARDS

## Abstract

Severe pneumonia-associated lung injury is a major cause of acute respiratory failure, acute respiratory distress syndrome (ARDS), and death in critically ill patients. Its course is shaped within the critically ill host environment by pulmonary infection and tissue injury, systemic inflammation, circulatory and organ dysfunction, host reserve, and ICU treatment exposures, and may be accompanied by changes in gut microbiota, barrier integrity, and cellular immune metabolism. Thus, pulmonary, intestinal, circulatory, and metabolic changes should be viewed as interrelated processes occurring at different physiological levels rather than as a predefined linear pathway. Emerging studies suggest links among gut microbiota remodeling, barrier disruption, microbial or host-derived circulating signals, altered microbial metabolites, and immune-cell metabolic reprogramming. However, their biological significance varies. Some may represent parallel responses to severe infection and critical illness, whereas others may arise secondary to lung injury, hypoxia, hypoperfusion, or treatment exposure. Longitudinal human evidence remains insufficient to establish whether gut-derived signals achieve physiologically effective remote exposure and independently modify lung injury. Mechanical ventilation is essential for severe respiratory failure, and its direct mechanical contribution to ventilator-induced lung injury has a relatively clear physiological basis. In contrast, sedation, hemodynamic and splanchnic perfusion changes, oxygenation and ventilation status, antibiotics, and nutritional support are distinct concomitant exposures that may independently influence gastrointestinal function, microbiota, barrier integrity, and immune metabolism. Using the critically ill host response as the overarching framework, this review evaluates the temporality, directionality, and functional support of relationships among gut microbiota, barrier function, candidate remote signals, cellular immune metabolism, and ventilation-related exposures, and assesses whether current evidence supports biologically and clinically meaningful gut-lung-immune-metabolic interactions. It also discusses lung-protective ventilation, nutritional support, microbiota modulation, anti-inflammatory therapy, and barrier protection. Current evidence derives mainly from limited studies in severe pneumonia or pneumonia-associated ARDS, clinical evidence from other critically ill populations, and preclinical studies. Evidence that pulmonary and systemic critical illness responses affect the gut is stronger than evidence for an independent gut-to-lung effect. Future longitudinal multi-compartment studies, source tracing, multi-omics integration, and mechanism-oriented interventions are needed to clarify functional cross-compartment relationships and their incremental clinical value for risk stratification and targeted treatment.

## 1 Introduction

Severe pneumonia is a major cause of acute respiratory failure, acute respiratory distress syndrome (ARDS), and critical illness-related death. Its core pathological processes include alveolar epithelial and pulmonary microvascular endothelial barrier injury after pathogen invasion, alveolar exudation, inflammatory cell infiltration, and oxygenation impairment (1, 2). SARS-CoV-2, influenza virus, respiratory syncytial virus, and various bacterial pathogens can all induce severe pneumonia, which rapidly progresses to ARDS and multiple organ dysfunction in some patients (3). Traditional research has primarily explained disease progression from perspectives such as local pulmonary inflammation, pathogen clearance, alveolar-capillary barrier disruption, and ventilator-induced lung injury (4). However, increasing evidence suggests that severe pneumonia is not merely a localized pulmonary infectious disease but a systemic pathological process accompanied by systemic inflammatory response, immune response remodeling, metabolic changes, and distant organ injury (5). Its evolution is also influenced by microbial load, host reserve, circulatory and microcirculatory status, and various critical care treatment exposures. Therefore, it is necessary to understand the dynamic connections between the lungs and extrapulmonary organs within the overall context of the critically ill host.

In recent years, the gut-lung axis has provided a new mechanistic perspective for understanding the potential microecological, immune, and metabolic connections between the intestine and the respiratory system. The gut microbiota can influence host defense and tissue function through changes in microbial composition and metabolites, regulation of barrier function, and immune responses. In critical illness, antibiotic exposure, hypoperfusion, hypoxia, fasting or insufficient enteral nutrition, sedation and analgesia, and systemic inflammatory response can all contribute to gut microbiota remodeling, characterized by decreased microbial diversity, reduced commensal bacteria, and enrichment of potentially pathogenic bacteria (6, 7). Concurrently, an impaired intestinal barrier may facilitate the translocation of microbial-associated components and host damage signals into the circulation, affecting systemic immunity and distant organ function (8–10). In severe pneumonia, these changes may be secondary to pulmonary infection, systemic inflammation, and treatment exposure, and may also, at specific stages, conversely modulate pulmonary responses through microbial, metabolic, or immune signals. Therefore, the “gut-lung axis” better encapsulates the time-dependent, potentially bidirectional, and asymmetrically supported interaction between the lungs and the intestine, rather than implying a single, fixed-direction gut-to-lung pathway. Currently, there is more direct physiological and clinical evidence for pulmonary infection and systemic critical illness secondarily affecting the intestine, while the comprehensive body of human evidence regarding gut-derived signals subsequently altering the course of lung injury remains relatively limited.

Immune-metabolic reprogramming represents the adaptive response of cells and intracellular processes to changes in infection, hypoxia, inflammation, mechanical stress, and the nutritional environment, rather than being an independent anatomical compartment parallel to the gut or lungs. Short-chain fatty acids (SCFAs), bile acids, and tryptophan metabolites can regulate macrophages, neutrophils, Treg/Th17 balance, and epithelial barrier repair; hypoxia and inflammatory stimuli may be accompanied by HIF-1α stabilization, enhanced glycolysis, lactate fluctuations, reactive oxygen species generation, and mitochondrial dysfunction (11). These metabolic changes can be influenced by the gut microecology or directly induced by infection, hypoxia, inflammation, circulatory abnormalities, and therapeutic stress, and thus should not be simply considered as an obligatory intermediary connecting the gut and lungs. During infection, leukocyte recruitment and redistribution, cytokine changes, and metabolic program switching can themselves be host defense responses adapted to the pathogen challenge, and do not necessarily have pathological significance simply because they deviate from a healthy resting state. Their biological effects depend more on the intensity, duration, cell type, and disease stage of the response; it is when this response no longer matches the continuously changing pathogen load or tissue demands, and is accompanied by barrier disruption, tissue damage, or organ dysfunction, that it more closely resembles a dysregulated or maladaptive response. From a biological organizational hierarchy perspective, the gut microbiome, intestinal barrier, immune metabolism, systemic inflammation, and lung injury correspond to ecological, tissue, cellular/intracellular, organismal, and tissue/organ-level processes, respectively. Connections between different levels may occur through microbial products, metabolites, inflammatory mediators, circulating cells, and neuro-humoral signals, but an abnormality at one level does not directly imply a corresponding functional change at another level. Therefore, the “gut-lung-immune-metabolic axis” in this article is primarily used to summarize the potential dynamic connections between these different levels, and its functional significance depends on whether cumulative evidence supports the signal source, effective exposure, target cell response, and organ effect.

Mechanical ventilation is a crucial life support measure for patients with severe pneumonia complicated by respiratory failure. Suboptimal settings for tidal volume, driving pressure, plateau pressure, positive end-expiratory pressure (PEEP), or excessive mechanical power can induce ventilator-induced lung injury (VILI), exacerbating alveolar epithelial and vascular endothelial damage, and promoting the release of DAMPs and inflammatory spillover (12, 13). At the same time, patients receiving mechanical ventilation often experience sedation and immobilization, increased intrathoracic pressure, hemodynamic changes, alterations in splanchnic perfusion, antibiotic exposure, adjustments in enteral nutrition, and fluctuations in oxygenation or carbon dioxide. These factors can all affect gastrointestinal motility, intestinal barrier integrity, microecology, and immune-metabolic status (14). Therefore, the direct pulmonary mechanical effects of mechanical ventilation should be distinguished from its accompanying circulatory, pharmacological, nutritional, and metabolic exposures; the latter are more likely to act as parallel modulating factors within the critically ill host environment, collectively shaping pulmonary, intestinal, and systemic responses, rather than constituting a single “mechanical ventilation-intestinal injury-lung injury” linear hierarchy. The effects of related immune and metabolic changes are also influenced by ventilation intensity, oxygenation status, circulatory reserve, and disease stage.

Previous reviews have separately summarized the gut-lung axis and microecological imbalance, infection-related immune-metabolic reprogramming, and VILI and lung-protective ventilation, but these areas are typically discussed in relative isolation. Existing research rarely integrates primary pulmonary infection, host systemic response, and time-varying factors such as mechanical ventilation, hemodynamics, sedation, antibiotics, and nutrition within a unified analytical framework. This article characterizes the nature of relationships supported by current evidence, examining whether the connections between intestinal, pulmonary, circulatory, and cellular immune-metabolic changes transcend temporal, source, and functional associations driven solely by common disease severity and treatment exposures. By comparing direct human evidence in severe pneumonia or pneumonia-associated ARDS, indirect evidence from other critically ill populations, and preclinical mechanistic studies, we further assess whether the existing evidence supports disease- or treatment-related parallel changes, directional cross-organ effects, or cross-organ networks with functional interdependence and incremental clinical value. This evidence-based evaluation delineates the current biological and clinical boundaries of the gut-lung-immune-metabolic connection and provides a basis for future longitudinal multi-compartment research and mechanism-stratified interventions.

## 2 Methods

### 2.1 Literature search strategy and eligibility criteria

The literature search was conducted in PubMed and the Web of Science Core Collection, covering the period from the inception of each database to August 2026, and was limited to English-language publications. Search terms were organized into four thematic groups: (1) Diseases and clinical scenarios, including “severe pneumonia,” “critical pneumonia,” “pneumonia,” “acute lung injury,” “acute respiratory distress syndrome,” “ARDS,” “critical illness,” and “sepsis”; (2) Gut-lung axis and intestinal abnormalities, including “gut–lung axis,” “gut microbiota,” “intestinal microbiome,” “dysbiosis,” “intestinal barrier,” “gut permeability,” “bacterial translocation,” and “microbial metabolites”; (3) Immunometabolic mechanisms, including “immunometabolism,” “metabolic reprogramming,” “HIF-1α,” “glycolysis,” “lactate,” “mitochondrial dysfunction,” “mitochondrial DNA,” “reactive oxygen species,” “NLRP3,” and “inflammasome”; (4) Mechanical ventilation and related therapeutic exposures, including “mechanical ventilation,” “ventilator-induced lung injury,” “VILI,” “mechanical power,” “sedation,” “antibiotics,” and “enteral nutrition.” Keywords and their synonyms within these thematic groups were combined using Boolean operators (“AND” and “OR”). Additionally, the reference lists of included studies and relevant high-quality reviews were manually screened to identify additional eligible studies, supplementing any potentially missed important studies from the database search and maximizing coverage. All authors participated in the literature screening and content organization.

### 2.2 Inclusion and exclusion criteria

Inclusion criteria were based on study context, biological domains, outcomes, and evidence type. Eligible studies included: (1) investigations involving severe pneumonia, pneumonia-associated acute lung injury or ARDS, sepsis, mechanical ventilation, or other relevant critical illness settings; (2) assessment of gut microbiota, intestinal barrier integrity, microbial metabolites, circulating PAMPs/DAMPs, systemic inflammation, immunometabolic reprogramming, or mitochondrial dysfunction; and (3) physiological, mechanistic, associative, prognostic, or interventional evidence relevant to lung injury, VILI, ventilator-associated pneumonia (VAP), multiple organ dysfunction syndrome (MODS), or other clinically meaningful outcomes. Human studies, animal models, and cell or ex vivo experiments were included. Systematic reviews, meta-analyses, guidelines, and expert consensus were used primarily for background synthesis and clinical contextualization rather than as substitutes for primary evidence.

Exclusion criteria included: (1) studies without substantive relevance to the review questions; (2) reports mentioning related concepts without empirical or mechanistic support; (3) conference abstracts, duplicate publications, or substantially overlapping datasets; (4) studies with insufficient methodological or outcome information; and (5) editorials, letters, news articles, or general commentaries without substantive evidence. Because direct evidence from severe pneumonia or pneumonia-associated ARDS remains limited, selected studies from sepsis, trauma, shock, intestinal ischemia–reperfusion, other mechanically ventilated populations, and relevant chronic inflammatory diseases were included when they provided transferable mechanistic insights. These studies were treated as indirect evidence rather than equivalent to severe pneumonia-specific data. Their applicability was interpreted cautiously according to disease context, timing, treatment exposure, tissue compartment, and experimental model.

### 2.3 Evidence evaluation

This paper adopts a problem-oriented evidence evaluation framework to examine the co-occurrence, temporal sequence, source and direction, functional dependence, and clinical relevance of candidate connections among the gut, lung, circulation, and host immune metabolism. It concurrently accounts for the impact of disease severity, shock, organ function, and treatment exposures such as mechanical ventilation, antibiotics, and nutrition. For mechanisms spanning different biological levels, further evaluation is conducted to determine whether there is coherent evidence linking signal sources, effective exposure, target cell response, and tissue effects. Based on the nature of the relationships supported by existing evidence, the connections discussed in this paper are classified into three categories: (1) Relationships supported by relatively direct physiological or functional evidence, i.e., those with clear physiological basis, temporal or functional links between relevant factors; (2) Cross-compartment or cross-level associations, i.e., correlations between different biological compartments observed in population studies, but whose temporal sequence, directionality, or functional dependence have not been fully established; (3) Candidate causal connections, i.e., potential pathways primarily supported by preclinical mechanisms, indirect clinical evidence, or biological plausibility, but lacking continuous human evidence for validation. Cross-compartment associations or mechanistic feasibility alone are insufficient to identify a relationship as an established functional cross-organ mechanism. Distinct from the above classification of “relationship nature,” this paper further categorizes studies into four types based on the directness of evidence sources: Type 1 includes direct human evidence from patients with severe pneumonia or pneumonia-associated ARDS; Type 2 includes clinical evidence from populations characterized by sepsis, receipt of mechanical ventilation, and other critically ill conditions; Type 3 includes preclinical evidence from animal, cellular, and ex-vivo experiments; Type 4 includes indirect evidence supporting biological plausibility from other disease contexts. When forming conclusions, priority is given to the directness of the disease context, temporal information, control of confounding factors, functional validation, and consistency of results, with judgment made by combining the two dimensions of “evidence source” and “relationship nature.” Statistical associations in patients with severe pneumonia do not automatically imply causal significance due to their direct source; conversely, a comprehensive mechanistic chain in experimental studies cannot be directly extrapolated as a clinical cross-organ mechanism in the absence of patient-level evidence on temporality, source, and effective exposure. Changes at one level also do not automatically represent corresponding functional changes at other biological levels. For mechanisms lacking specific human support in severe pneumonia, this paper uses expressions such as “may be involved,” “candidate connection,” or “candidate mechanism”; intervention strategies primarily supported by preclinical or indirect evidence are positioned as exploratory directions.

## 3 Pathological basis of lung injury associated with severe pneumonia

### 3.1 Pulmonary infection, alveolar epithelial injury, and endothelial barrier disruption

The core pathological basis of lung injury associated with severe pneumonia is the disruption of the alveolar epithelial and pulmonary microvascular endothelial barriers. Pathogen-associated molecular patterns (PAMPs) and damage-associated molecular patterns (DAMPs) released by damaged cells are recognized by alveolar macrophages, alveolar epithelial cells, and endothelial cells, which activates TLR/NF-κB, inflammasomes, and related pro-inflammatory pathways, and induces the release of inflammatory cytokines such as TNF-α, IL-1β, and IL-6 (15, 16). This process plays a crucial role in host antimicrobial defense but may also promote the amplification of alveolar inflammation when this response becomes dysregulated.

Neutrophil infiltration is an important pathological event in the progression of lung injury. Although essential for pathogen clearance, these mediators released by activated neutrophils simultaneously damage alveolar epithelial cells, microvascular endothelial cells, and intercellular junctions, thereby reducing the integrity of the alveolar-capillary barrier (17, 18). Although different pathogens have distinct virulence mechanisms, severe lung injury typically manifests as increased alveolar permeability, proteinaceous exudation, pulmonary edema, decreased lung compliance, impaired oxygenation, and progression to ARDS (19, 20). Additionally, activation of the complement and coagulation systems may further aggravate lung injury by promoting neutrophil chemotaxis, endothelial hyperpermeability, microthrombus formation, and microcirculatory disturbances (21). Thus, pulmonary immune-inflammatory imbalance, disruption of the alveolar-capillary barrier, and microcirculatory disturbances constitute a relatively clear pathological basis for ALI/ARDS associated with severe pneumonia (22). However, evidence regarding specific mechanisms such as NETs, inflammasomes, complement, and microthrombi is partly derived from COVID-19-associated pneumonia, other types of ARDS, or experimental models, and their relative roles in severe pneumonia caused by different pathogens require further clarification.

### 3.2 From local lung injury to systemic critical host response

The spillover of pulmonary inflammation has received some support from clinical studies of ARDS and other critical illnesses, but evidence that it further exacerbates lung injury through intestinal damage primarily comes from other critical care studies and experimental models. When local inflammation exceeds the regulatory capacity of the lungs, the damaged alveolar-capillary barrier may become a major channel for inflammatory mediators to enter the circulation (23). DAMPs such as inflammatory cytokines, NETs, mtDNA, and HMGB1 released by alveolar macrophages, neutrophils, and damaged epithelial and endothelial cells can activate different pattern recognition and nucleic acid sensing pathways (24). Among these, mtDNA may induce inflammation and type I interferon responses through endosomal TLR9 or the cytosolic cGAS-STING-TBK1-IRF3 signaling pathway, and together with RAGE, NLRP3 inflammasome, complement, and coagulation systems, influence systemic inflammation and distant organ injury (25, 26). Thus, lung injury associated with severe pneumonia may extend from a local infectious lesion to a systemic process involving immune, coagulation, and microcirculatory abnormalities.

In this process, systemic inflammation, hypoperfusion, and hypoxia may impair the intestinal barrier, heightening the risk of gut-derived PAMPs/DAMPs entering the circulation (27). Meanwhile, coagulation activation and microthrombus formation may further compromise microcirculatory perfusion in organs such as the lungs, intestines, and kidneys (28). Additionally, animal and other disease models suggest that immune cells derived from or regulated by intestinal signals may participate in pulmonary immune responses, with some models observing the recruitment of γδ T17 cells to the lungs and production of IL-17A. However, IL-17A-related responses are highly context-dependent: moderate activation can promote neutrophil recruitment and clearance of extracellular pathogens, while sustained or excessive activation, especially when Treg/Th17 balance is disrupted, may drive persistent inflammation and barrier damage. The net effect depends on the pathogen type, cell source, response intensity, and disease stage (29). Therefore, current evidence only supports the biological possibility of gut-related immune signals participating in pulmonary regulation, but cannot prove that gut-derived γδ T17 cell migration is a universal pro-inflammatory driving mechanism for lung injury in severe pneumonia, nor can it confirm that the “pulmonary inflammation spillover-intestinal barrier injury-gut-derived signals re-entering the circulation-further aggravation of lung injury” constitutes a continuous causal pathway in patients.

As lung injury progresses to a systemic critical condition, systemic inflammation, hypoxia, microcirculatory disturbances, hemodynamic changes, and organ support therapies often simultaneously affect multiple physiological compartments. The resulting intestinal, metabolic, and immune abnormalities may reflect the impact of pulmonary disease on distant organs, or may be parallel changes caused by disease severity and common treatment exposures; in some contexts, these changes may further participate in cross-organ feedback. Therefore, the lung–gut connection in severe pneumonia is better understood within a dynamic critical host environment rather than summarized by a unidirectional continuous propagation process.

### 3.3 Dual effects of mechanical ventilation on lung injury

Mechanical ventilation is a crucial life support measure for maintaining oxygenation and ventilation in patients with severe pneumonia complicated by ALI/ARDS, but inappropriate ventilation intensity may also constitute a “second hit” beyond the primary infectious lung injury (30). Excessive tidal volume, driving pressure, plateau pressure, or mechanical power can cause alveolar overdistension, repeated collapse–recruitment, and shear stress injury, inducing or aggravating VILI (31, 32). In addition to direct mechanical damage, mechanical stress may also trigger biotrauma and spillover of inflammatory mediators, further extending local pulmonary inflammation into a systemic response (33, 34). There is robust clinical and experimental evidence supporting the direct damaging effects of VILI on lung tissue.

In contrast, the hypothesis that mechanical ventilation exacerbates lung injury via distant intestinal abnormalities is currently supported primarily by indirect evidence derived from other critical care clinical studies and animal experiments (35, 36). Patients undergoing mechanical ventilation typically experience simultaneous sedation and immobilization, hemodynamic changes, fluctuations in oxygenation and carbon dioxide, antibiotic exposure, and adjustments in enteral nutrition, all of which may individually or collectively affect gastrointestinal motility, visceral perfusion, microbiota, the intestinal barrier, and host immune-metabolic status. Therefore, the direct mechanical effects of mechanical ventilation on lung tissue should be distinguished from its accompanying systemic and treatment-related exposures. Current evidence suggests that mechanical ventilation and the associated therapeutic environment can simultaneously affect pulmonary mechanical stress, systemic inflammation, and distant organ function (37, 38). However, these changes are more likely to represent parallel actions and dynamic interactions of multiple factors in a critically ill host, rather than establishing a single causal hierarchy of “mechanical ventilation—intestinal abnormality—gut-derived signals—lung injury.” Thus, in subsequent discussions of intestinal microbiota, barrier function, and immune-metabolic mechanisms, three possible relationships need to be considered simultaneously: parallel changes caused by disease and treatment exposures, the impact of lung injury and systemic responses on the intestine, and the yet-to-be-verified feedback effects of gut-derived signals on the lungs. The relative contributions of these distinct pathways may vary with disease stage, circulatory status, and treatment exposure (**Figure 1**).

**Figure 1:**
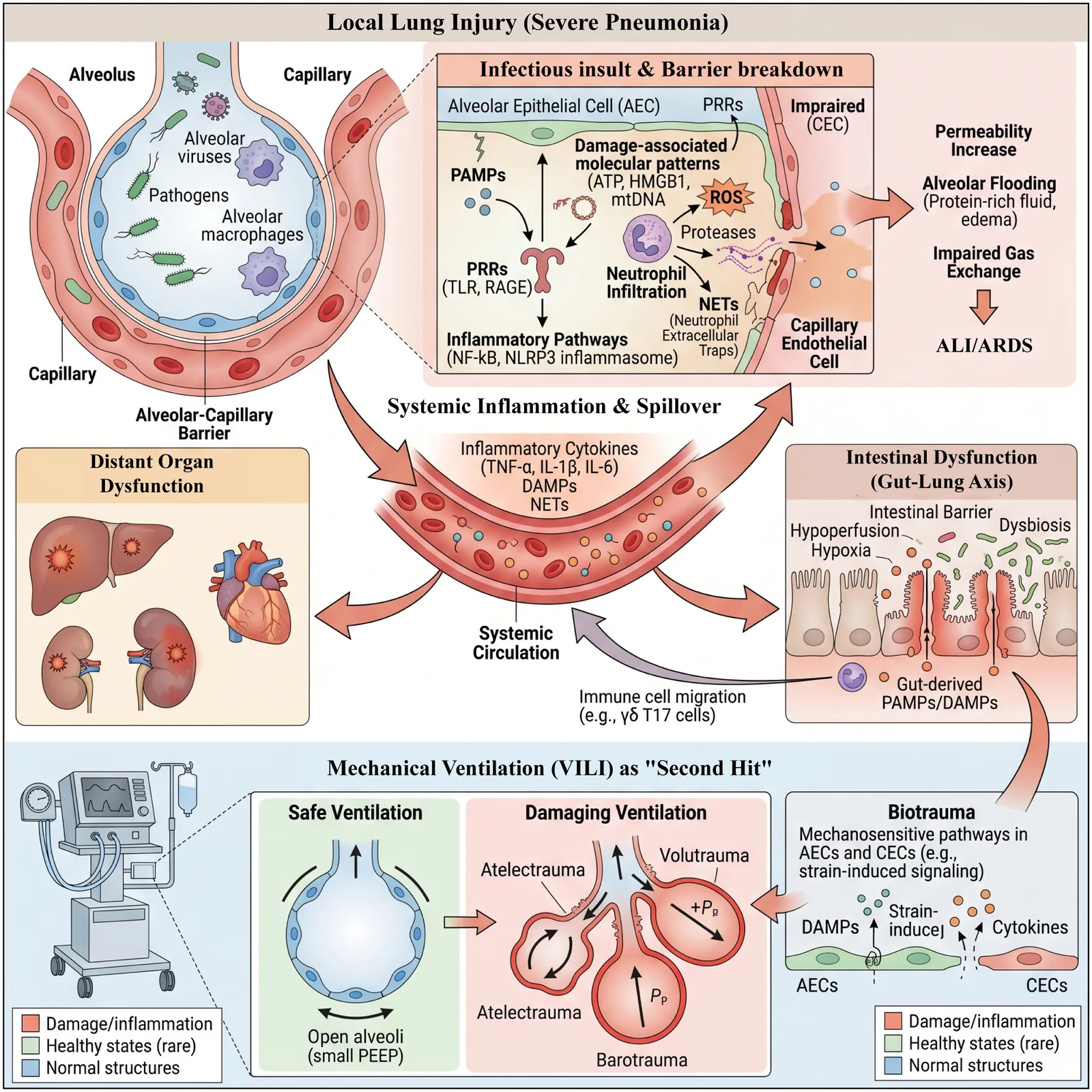
Local lung injury, systemic inflammatory spillover, and mechanical ventilation-related “second hit” in severe pneumonia. Pathogen infection disrupts the alveolar-capillary barrier, PAMPs/DAMPs activate PRRs and inflammatory pathways such as NF-κB and NLRP3, inducing ROS generation, neutrophil infiltration, NET release, and endothelial injury, leading to alveolar leakage, gas exchange impairment, and ALI/ARDS. Inflammatory cytokines, DAMPs, and NETs entering the circulation can trigger distant organ injury and amplify pulmonary inflammation through intestinal barrier disruption, dysbiosis, and gut-derived inflammatory signals. If mechanical ventilation parameters are inappropriate, it can further induce biotrauma through barotrauma, volutrauma, atelectrauma, and mechanosensitive signaling, constituting a “second hit” in the progression of severe pneumonia. (Solid black arrows indicate known or relatively clear pathological processes; thick orange curved arrows indicate the main propagation directions among lung injury, systemic circulation, distant organs, and the gut; solid purple arrows indicate candidate gut-lung interaction pathways such as immune cell migration; solid orange arrows in the mechanical ventilation panel indicate the progression or amplification effects of ventilation-related injury. AECs, alveolar epithelial cells; ALI, acute lung injury; ARDS, acute respiratory distress syndrome; ATP, adenosine triphosphate; CECs, capillary endothelial cells; DAMPs, damage-associated molecular patterns; HMGB1, high-mobility group box 1; mtDNA, mitochondrial DNA; NETs, neutrophil extracellular traps; NF-κB, nuclear factor-kappa B; NLRP3, NOD-like receptor family pyrin domain-containing 3; PAMPs, pathogen-associated molecular patterns; PEEP, positive end-expiratory pressure; PRRs, pattern-recognition receptors; RAGE, receptor for advanced glycation end products; ROS, reactive oxygen species; TLR, Toll-like receptor; TNF-α, tumor necrosis factor-alpha; VILI, ventilator-induced lung injury; γδ T17 cells, interleukin-17-producing gamma delta T cells).

## 4 Gut microbiome imbalance: candidate regulatory factors in severe pneumonia-associated lung injury

The gut microbiome alterations described in this chapter are primarily supported by studies conducted in general ICU settings, sepsis, and other critically ill populations, whereas direct longitudinal evidence specific to severe pneumonia or pneumonia-associated ARDS remains limited. Existing studies consistently suggest that critical illness is characterized by remodeling of gut microbiota composition and metabolic function, but this process is modulated by a confluence of factors including infection severity, circulatory status, host reserve, as well as antibiotics, nutrition, sedation, and organ support. Therefore, this chapter focuses on examining which reproducible changes occur in the gut microbiome during critical illness, their temporal dynamics in the course of severe pneumonia, and whether they have functional implications for pulmonary outcomes beyond disease severity and treatment exposure.

### 4.1 Rapid remodeling of gut microbiota in the ICU environment

Studies from general ICU, sepsis, and other critically ill populations indicate that the gut microbiome can undergo significant remodeling rapidly during critical illness, primarily characterized by decreased microbial diversity, reduced commensal bacteria, and enrichment of opportunistic pathogens; however, specific longitudinal evidence in severe pneumonia patients remains limited (39, 40). While broad-spectrum antibiotics suppress pathogenic load, they can also non-selectively reduce intestinal commensal bacteria, weaken colonization resistance, and facilitate the proliferation of potential pathogens such as Enterobacteriaceae and Enterococcus (41, 42). Factors such as fasting or delayed enteral nutrition, sedation and analgesia, decreased gastrointestinal motility, shock, hypoxia, and intestinal hypoperfusion may also collectively affect gut homeostasis. Thus, ICU-associated gut dysbiosis is typically a result of the combined effects of disease severity and multiple treatment exposures. Current human evidence supports significant gut microbiome remodeling during critical illness, but in severe pneumonia, its timing and independent contribution beyond antibiotics, nutrition, mechanical ventilation, and disease severity remain unclear. Therefore, it is more appropriate to regard it as a dynamically formed microbial phenotype in critical illness rather than a predetermined initiating factor of the disease.

### 4.2 Changes in gut microbiota metabolites

Gut microbiota remodeling is often accompanied by changes in microbial metabolic functions, with SCFAs, bile acids, and tryptophan metabolites receiving considerable attention. Relevant functional evidence mainly comes from animal and cell studies or other disease contexts, and the dynamic changes and direct effects of these metabolites in severe pneumonia patients have not been fully validated (43, 44). SCFAs primarily include acetate, propionate, and butyrate, produced by gut commensal bacteria fermenting dietary fiber; among them, butyrate and propionate are closely related to intestinal epithelial energy metabolism, mucosal barrier maintenance, and immune homeostasis regulation. In addition to local effects in the gut, SCFAs can regulate immune cell function through signaling via receptors such as FFAR2, FFAR3, and GPR109A, as well as inhibition of histone deacetylases (HDACs) (45). Some SCFAs that enter the circulation may also affect the differentiation and functional programming of bone marrow hematopoietic progenitors and myeloid precursor cells, thereby altering the output of immune cells such as monocytes, neutrophils, and dendritic cells. This provides a potential systemic pathway for gut microbial metabolites to remotely regulate lung immunity (46).

During critical illness, a reduction in SCFAs-producing commensal bacteria may lead to decreased SCFAs production capacity, but actual levels in patients are also influenced by multiple factors including dietary fiber and enteral nutrition intake, gastrointestinal motility, intestinal absorption, drug exposure, and liver and kidney metabolism and clearance (47). Therefore, from “changes in microbiota composition” to “changes in circulating metabolites” and then to “local receptor or cellular responses in the lungs,” this involves multiple steps including production, absorption, transport, metabolic clearance, and target tissue exposure. A decrease in the abundance of SCFAs-producing bacteria cannot directly represent a reduction in circulating SCFAs, nor can it be used to infer corresponding changes in local lung signaling.

Gut microbiota also participate in the conversion of primary bile acids to secondary bile acids and influence the bile acid profile and FXR and TGR5-related signaling (48, 49). However, bile acid signaling is highly context-dependent, and its effects may be influenced by disease stage, bile acid type, receptor expression, host metabolic status, and inflammatory background (50). Tryptophan can be metabolized into indole-based aryl hydrocarbon receptor (AhR) ligands or enter the kynurenine pathway; inflammation and gut dysbiosis may alter the balance between different metabolic pathways (51, 52). Overall, changes in SCFAs, bile acids, and tryptophan metabolism suggest that during critical illness, the gut microbiome undergoes not only compositional changes but also functional remodeling. However, these metabolites are simultaneously affected by nutritional intake, intestinal absorption, liver and kidney function, inflammation severity, and drug exposure. Current human studies are insufficient to establish a continuous temporal relationship of “microbiota changes—circulating metabolite exposure-pulmonary cellular responses,” so these metabolites are more appropriately considered candidate regulatory signals, and their independent functional contributions in severe pneumonia remain to be defined.

### 4.3 Gut-derived pathogen translocation and risk of secondary infection

Gut microbiome imbalance may influence the risk of secondary infection by weakening colonization resistance, promoting opportunistic pathogen enrichment, and increasing microbial translocation opportunities (53). Under normal conditions, gut commensal bacteria limit the overgrowth of opportunistic pathogens such as Klebsiella, Escherichia coli, and Enterococcus through ecological niche competition, metabolite-mediated inhibition, and immune regulation (54). However, under conditions of broad-spectrum antibiotic use, immunosuppression, hypoperfusion, and barrier damage, this protective effect may be diminished, allowing potential pathogens to enrich within the gut (55).

Whole-genome comparative studies of bacterial strains in some ICU and ventilator-associated pneumonia (VAP) patients suggest a possible source link between gut strains and lung infection strains, providing preliminary clues for “gut-lung microbiota migration” (56). However, such studies are mostly case reports or small sample analyses, and their frequency, transmission direction, and independent contribution relative to oropharyngeal, airway colonization, and environmental sources remain insufficiently evaluated. Therefore, strain concordance currently mainly provides source-tracking clues and is insufficient to prove that the gut is a universal source of pathogens in severe pneumonia or VAP.

Additionally, evidence from other diseases and critical illness, such as stroke and trauma, suggests that intestinal barrier damage and immune imbalance may promote the entry of bacteria or their products into the circulation and are associated with increased susceptibility to lung infection (57). Animal studies further indicate that bacteria, fungi, or their components can undergo remote dissemination after barrier damage or affect lung infection status (58, 59). However, crossing the intestinal barrier, entering the circulation, reaching the lungs, and exerting biological effects in the lungs are distinct processes. Even if microbial translocation or the translocation of their products is confirmed, it must be further demonstrated that they can evade first-pass hepatic processing and systemic clearance, reach the lungs, and achieve effective exposure levels sufficient to influence pathogen colonization, infection susceptibility, or inflammatory responses locally (60). The above studies mainly indicate the biological feasibility of remote microbial or related signal transmission, but since the primary disease, initial injury site, and pathological timing are not entirely consistent with severe pneumonia, their results cannot be directly extrapolated to gut-derived pulmonary effects in severe pneumonia.

Overall, existing evidence strongly supports rapid gut microbiome remodeling during critical illness and suggests a possible source link between the gut and infection strains in some patients. However, in severe pneumonia, there is a lack of sufficient longitudinal human evidence to demonstrate that these changes precede lung injury deterioration, are independent of disease severity and treatment exposure, or consistently produce effective remote signals capable of altering lung responses. Therefore, current evidence supports critical illness-associated microbiome remodeling and candidate abscopal effects, rather than an established gut-derived lung injury mechanism. Future research requires multi-compartment longitudinal sampling of feces, blood, and airways, strain-level source tracking, treatment exposure adjustment, and local effective exposure assessment to further clarify the temporal sequence and clinical contribution (**Figure 2**).

**Figure 2:**
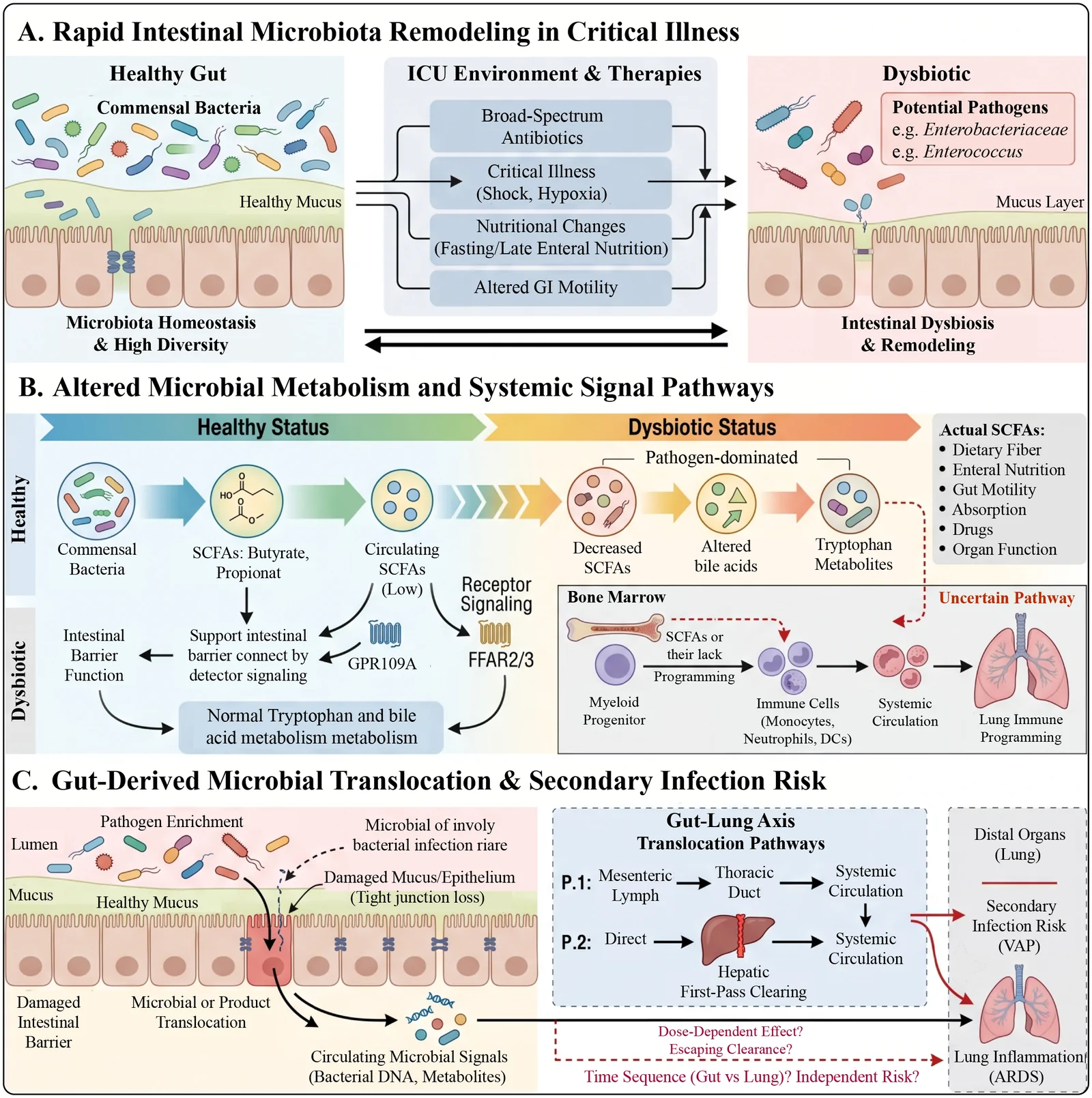
Gut microbiome remodeling, metabolic changes, and candidate abscopal connections in critical illness. **(A)** Critical illness, ICU treatment, broad-spectrum antibiotics, altered nutrition, and gastrointestinal motility abnormalities can collectively drive the gut microbiota from a relatively stable microecological state to critical illness-associated microbiota remodeling, characterized by reduced commensal bacteria, damaged mucus layer, and enrichment of potential pathogens. **(B)** Gut dysbiosis is accompanied by reduced SCFAs levels, abnormal bile acid and tryptophan metabolism, and may affect lung immunity through GPR109A, FFAR2/3, bone marrow immune programming, and circulating immune cells. **(C)** After intestinal barrier damage, microorganisms and their products may enter the circulation and represent candidate pathways for remote exposure via the portal vein-liver, mesenteric lymphatics, or systemic circulation; their source, effective dose, temporal sequence, and pulmonary functional effects remain to be validated. (Solid black arrows indicate well-established biological processes; bidirectional arrows indicate mutual influences; gradient arrows indicate the continuous transition from health to gut dysbiosis; solid red arrows indicate candidate pathways for gut-derived signals to the lungs; red dashed arrows and question marks indicate hypotheses yet to be verified. Different shapes and colors of bacteria represent different microbial taxa; red, green, and blue backgrounds indicate damage/inflammation, relative health, and normal structure, respectively.).

## 5 Intestinal barrier injury and the candidate lung-gut distant connection

Intestinal barrier abnormalities in severe pneumonia are supported by limited evidence in critically ill patients, but the barrier injury itself and its potential distant effects need to be evaluated separately. Existing research supports that systemic inflammation, hypoxia, hypoperfusion, and treatment exposure can be accompanied by intestinal barrier damage, but there is relatively limited evidence supporting whether these resulting microbial or host signals can effectively reach the lungs and alter the trajectory of lung injury. This chapter accordingly discusses intestinal barrier injury, candidate circulatory signals, and their possible gut-to-lung axis.

### 5.1 Intestinal barrier disruption: from tight junction injury to potential distant signal release

Direct dynamic studies of the intestinal barrier in patients with severe pneumonia or pneumonia-associated ARDS remain scarce, with current understanding mainly derived from sepsis, other critical illness clinical studies, and animal models. In the context of severe pneumonia, intestinal barrier abnormalities are more likely secondary to systemic inflammation, hypoxia, shock, splanchnic hypoperfusion, and exposure to mechanical ventilation and related treatments caused by pulmonary infection, rather than being an upstream event established prior to lung injury (61, 62). Under conditions of systemic inflammation, hypoperfusion, and hypoxia, the energy supply to intestinal epithelial cells may be impaired, and mitochondrial dysfunction and ATP depletion can lead to abnormal expression and distribution of tight junction proteins, thereby increasing intestinal mucosal permeability (62, 63). At the same time, inflammatory factors such as TNF-α and IFN-γ can promote the endocytosis, degradation, and disassembly of intercellular junctions of tight junction proteins through pathways such as myosin light chain kinase and Rho kinase (64).

Intestinal barrier injury may also be accompanied by increased epithelial cell apoptosis, reduced mucosal repair capacity, and weakened local defense function (65). After the integrity of the barrier declines, bacteria and their structural components, endotoxins, and host damage signals may cross the intestinal barrier and enter the portal vein or mesenteric lymphatic system (66). However, signals transmitted via the portal vein must undergo hepatic first-pass processing and systemic clearance, while the main evidence for mesenteric lymphatic transmission comes from preclinical models with significant intestinal ischemia and barrier disruption (67). Therefore, increased barrier permeability primarily indicates impaired defense function at the tissue level and does not necessarily mean that relevant signals will inevitably enter the systemic circulation or reach the lungs. Sepsis and acute lung injury studies show that intestinal barrier dysfunction often coexists with microbial translocation and enhanced systemic inflammation (68). However, this co-occurrence is still influenced by circulatory disorders, disease severity, and treatment exposure. Nevertheless, existing human and preclinical evidence supports that critical illness can be accompanied by intestinal barrier injury, but in severe pneumonia, its occurrence is usually concurrent with systemic inflammation, hypoxia, hypoperfusion, and treatment exposure. At present, the barrier abnormality itself is relatively clear, but whether it precedes the worsening of lung injury and constitutes an independent driver of lung function remains uncertain.

### 5.2 Gut-derived PAMPs/DAMPs and candidate inflammatory propagation networks

After intestinal barrier damage, PAMPs such as LPS, bacterial DNA, and peptidoglycan, as well as DAMPs released from intestinal epithelial injury, may enter the portal vein or lymphatic system; however, whether they can maintain biological activity and reach distant target organs depends on subsequent clearance, metabolism, and tissue uptake processes (69, 70). LPS can form complexes with LPS-binding protein (LBP), activate the TLR4/NF-κB pathway upon presentation by CD14, thereby potentially disseminating inflammatory stimuli associated with intestinal barrier abnormalities throughout the systemic circulation (71, 72). Experimental studies also suggest that PAMPs/DAMPs may activate the NLRP3 inflammasome, promoting the maturation and release of IL-1β and IL-18; activation of the complement and coagulation systems may further promote leukocyte chemotaxis, endothelial injury, increased vascular permeability, and microcirculatory disturbances (73, 74). In addition to TLR and inflammasome pathways, cytosolic host or microbial DNA may be recognized by cyclic GMP-AMP synthase (cGAS), inducing type I interferon and related inflammatory responses through the STING-TBK1-IRF3 signaling pathway (75). These pathways have clear molecular biological foundations, but the tissue origin of circulatory signals and their actual degree of activation in specific distant target cells remain to be clearly distinguished.

Anatomically, gut-derived microbial components and host damage signals transmitted via the portal vein first reach the liver, where their concentration, structure, and biological activity may be significantly affected by Kupffer cell phagocytosis, hepatocyte metabolism, and clearance by the hepatic reticuloendothelial system. Only signals that escape or exceed the liver’s clearance capacity can further enter the systemic circulation (76). Therefore, increased intestinal barrier permeability or detection of related molecules in peripheral blood does not equate to their ability to reach the pulmonary circulation at biologically effective concentrations. Even if relevant signals are present in the peripheral circulation, it still needs to be demonstrated that they can enter the pulmonary vasculature or alveolar compartment, be taken up by lung target cells, and activate corresponding pathways. From “gut origin” to “circulatory presence,” then to “effective lung exposure” and “local pathway activation,” this involves multiple consecutive but independent biological steps. There is currently a lack of longitudinal evidence linking gut origin, hepatic first-pass processing, peripheral circulation concentration, local lung exposure, and intracellular pathway activation in patients with severe pneumonia.

The nature of evidence provided by different models varies. Studies on pneumonia or pneumonia-associated ARDS suggest that circulatory PAMPs/DAMPs, inflammasomes, nucleic acid sensing signals, and complement-coagulation abnormalities may be associated with disease severity, but their gut origin or temporal sequence cannot usually be confirmed (77, 78). Sepsis and general ARDS studies can support an indirect association between intestinal barrier abnormalities, systemic inflammation, and organ injury, but due to heterogeneity in infection sources and etiology, they cannot directly prove the same causal sequence in severe pneumonia (79). Models featuring intestinal hypoperfusion or circulatory injury as primary initiating events primarily illustrate the biological plausibility of gut-derived signals affecting the lungs, serving as analogical evidence. Cell and animal studies can explain the potential roles of TLR4/NF-κB, NLRP3, cGAS-STING, complement, and coagulation systems, but cannot prove that these pathways constitute a complete and directionally defined transmission chain in patients with severe pneumonia (80). Based on existing research, PAMPs/DAMPs and pathways such as TLR4/NF-κB, NLRP3, and cGAS-STING have sufficient biological feasibility, but there is still a lack of continuous evidence simultaneously linking “gut origin—systemic effective exposure—lung target cell response” in patients with severe pneumonia. Therefore, current evidence primarily supports candidate distant inflammatory mechanisms but cannot determine their origin, direction, and relative contribution in patients.

### 5.3 Candidate lung-gut bidirectional feedback: from secondary intestinal changes to potential lung effects

The lung-gut connection in severe pneumonia exhibits significant directional asymmetry. Clinical course and physiological evidence provide stronger support for the notion that pulmonary infection and systemic critical responses occur first, subsequently affecting the intestine through inflammation, hypoxia, hypoperfusion, and microcirculatory abnormalities, as well as treatment exposure; evidence for secondary intestinal abnormalities further altering lung injury in the reverse direction mainly comes from preclinical and other critical illness studies.

Pulmonary inflammation associated with severe pneumonia can promote the release of mediators such as cytokines, NETs, mtDNA, and HMGB1 into the circulation, potentially affecting intestinal epithelial cells, intestinal microcirculation, and microbiota status (81, 82). Among these, circulating mtDNA, after being taken up by target cells and entering endosomes or the cytoplasm, may participate in TLR9 or cGAS-STING-related nucleic acid sensing responses, but its cellular origin, uptake mode, and distant effects remain unclear (83). Changes in intrathoracic pressure and hemodynamics caused by mechanical ventilation, as well as exposure to sedation, immobilization, broad-spectrum antibiotics, delayed enteral nutrition, and vasoactive drugs, may also jointly affect gastrointestinal motility, splanchnic perfusion, microbiota composition, and barrier integrity (84). Therefore, intestinal abnormalities in severe pneumonia are likely to arise in the context of the combined effects of the primary lung disease, systemic host response, and treatment exposure, rather than being the result of a single lung-derived mediator.

In experimental or critical illness contexts with increased intestinal barrier permeability, LPS, bacterial DNA, and other microbial or host-derived signals may enter the blood or lymphatic system and, having escaped clearance while maintaining biological activity, reach lung target cells to affect pulmonary vascular endothelium, alveolar macrophages, and inflammatory cell recruitment (85). Among these, DNA taken up by lung cells and entering the cytoplasm may activate the cGAS-STING-TBK1-IRF3 signaling pathway, inducing type I interferon responses; however, detection of DNA in the circulation does not directly indicate its gut origin, nor does it equate to activation of the lung cGAS-STING pathway (86). Similarly, the entry of signals into the pulmonary circulation does not necessarily demonstrate that they can cross the pulmonary vascular-alveolar barrier or reach concentrations in the alveolar compartment sufficient to induce biological effects.

In addition to the blood circulation, the mesenteric lymphatic system may also constitute a potential signal transmission pathway. This route can partially bypass the portal vein and hepatic first-pass clearance and enter the systemic circulation via the thoracic duct, thereby offering an anatomically direct pathway for gut-derived signals to reach the pulmonary circulation (87). Related preclinical studies suggest that mesenteric lymph fluid can carry lipid mediators, microbial components, and host damage signals into the systemic circulation and is associated with distant pulmonary inflammatory responses (88). However, these pieces of evidence mainly come from models of trauma, hemorrhagic shock, intestinal ischemia-reperfusion, and other conditions with significant splanchnic hypoperfusion, where the degree of intestinal ischemia, extent of barrier disruption, and lymphatic signal output may be significantly stronger than in most patients with severe pneumonia where pulmonary infection is the initial event. Therefore, the mesenteric lymphatic pathway mainly provides anatomical and mechanistic feasibility evidence, and its occurrence intensity, time window, and relative contribution in severe pneumonia remain unclear.

Furthermore, TMAO-related metabolism, intestinal fungal overgrowth, and other microbial signals may also affect pulmonary inflammatory status through NLRP3 inflammasomes, alveolar macrophages, and immune cell migration (89). These studies support the anatomical and mechanistic possibility of gut-derived signals affecting the lungs, but have not yet demonstrated that relevant signals in patients with severe pneumonia can escape hepatic and systemic clearance, reach local lung compartments, and achieve a threshold concentration sufficient to alter the course of lung injury. The actual effects of these candidate mechanisms may also be modulated by a combination of pathogen type, circulatory status, degree of intestinal barrier injury, host immune status, and disease stage.

In population studies, D-lactate, diamine oxidase (DAO), and intestinal fatty acid-binding protein (I-FABP) are mainly used as candidate biomarkers reflecting intestinal mucosal permeability or intestinal epithelial injury. Some mixed critical illness cohorts show that these markers are associated with systemic inflammation, oxygenation impairment, lung injury severity, or poor prognosis (90). However, their elevation may also occur concurrently with shock, hypoperfusion, organ dysfunction, and overall disease severity. Therefore, these markers more directly reflect the severity of intestinal injury or critical illness, while their direct involvement in the progression of lung injury remains a distinct mechanistic question. Correlation itself cannot determine whether intestinal barrier abnormalities occur before the worsening of lung injury, nor can it prove that they independently mediate the progression of ARDS. Furthermore, these markers also cannot provide direct evidence that gut-derived signals escape hepatic clearance, reach the alveolar compartment, or activate lung target cells.

Overall, existing clinical course and physiological evidence provide stronger support for the notion that primary pulmonary infection and critical host responses can secondarily affect the intestine, while evidence for intestinal abnormalities further altering lung injury in the opposite direction is significantly weaker. Markers such as D-lactate, DAO, and I-FABP support the presence of intestinal barrier injury in patients but mainly reflect tissue injury or disease severity, and cannot yet determine the origin of gut-derived signals, effective lung exposure, or their independent functional effects. Therefore, the currently more established connection is “lung/systemic response → intestinal changes,” while “intestinal changes → lung functional effects” remains a candidate feedback. Simultaneous longitudinal sampling of the lungs, blood, and intestine, along with source tracing, will be key to distinguishing between the two (**Figure 3**).

**Figure 3:**
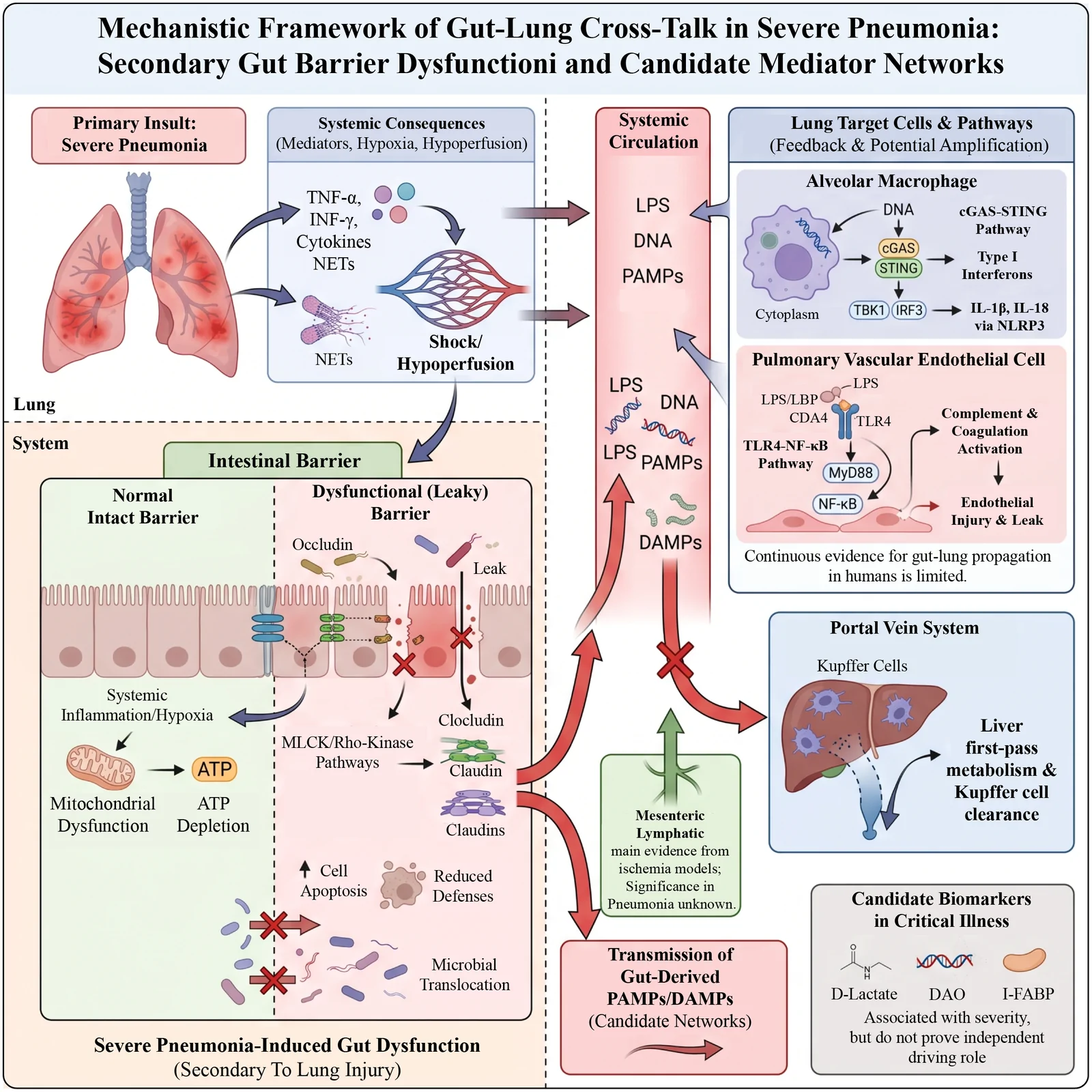
Directional changes in the lung-gut axis and candidate distant signals in severe pneumonia. Severe pneumonia can first affect the intestinal barrier and microbiota through systemic inflammation, hypoxia, hypoperfusion, circulatory changes, and treatment exposure. After secondary barrier injury, LPS, DNA, and other PAMPs/DAMPs may enter the systemic circulation via the portal vein-liver or mesenteric lymphatic pathway and, after forming effective lung exposure, act on alveolar macrophages and pulmonary vascular endothelial cells, involving candidate pathways such as TLR4-NF-κB, NLRP3, and cGAS-STING. Current evidence supports the secondary impact of lung/systemic responses on the intestine, while the temporal sequence, effective exposure, and independent functional contribution of gut-derived signals further altering lung injury remain insufficiently determined. D-lactate, DAO, and I-FABP can reflect intestinal barrier injury but cannot alone prove gut-derived lung injury mechanisms. (Black/dark blue solid arrows indicate relatively clear pathological processes; red solid arrows indicate gut-derived PAMPs/DAMPs entering the circulation and candidate transmission pathways; green arrows indicate the mesenteric lymphatic pathway, with limited evidence; blue arrows indicate the portal vein-liver clearance process; dashed lines distinguish different regions of the lung, systemic circulation, and intestine; red crosses indicate barrier disruption, pathway blockade, or unconfirmed causality; upward arrows indicate increased apoptosis.).

## 6 Immunometabolic reprogramming: a candidate integrative layer of lung, gut, and systemic host responses

Immunometabolic reprogramming in severe pneumonia can be induced by pathogens, hypoxia, inflammation, mechanical stress, circulatory abnormalities, and nutritional or drug exposure, and may also be modulated by gut-derived metabolic signals. It is neither synonymous with pathological dysregulation nor necessarily an intermediary between the gut and lungs. Metabolic shifts can support pathogen clearance, cell survival, and barrier repair, but may also promote inflammation or tissue damage under specific cellular and persistent stimulatory conditions. Therefore, this chapter focuses on evaluating the cell- and stage-dependent roles of different immunometabolic changes and whether existing evidence can establish functional links between specific gut-derived signals and pulmonary effects.

### 6.1 Cell- and stage-dependent roles of HIF-1α-related glycolysis

In the hypoxic, pathogen-stimulated, and inflammatory environment of severe pneumonia, alveolar macrophages, neutrophils, and alveolar epithelial cells may undergo metabolic adaptations (91). HIF-1α is a key transcription factor linking hypoxia sensing to metabolic regulation; its stabilization upregulates glucose transporters, glycolytic enzymes, and lactate dehydrogenase A, promoting a shift from oxidative phosphorylation to glycolysis (92). This metabolic shift primarily reflects cellular adaptation to changes in oxygen and energy supply-demand, contributing to ATP maintenance, cell survival, and local functional regulation, with net effects not inherently directed toward inflammation amplification or tissue damage. At the tissue level, these intracellular metabolic changes translate into pathophysiological outcomes only when they alter inflammatory mediator release, barrier function, cell survival, or the tissue microenvironment.

Significant heterogeneity exists across different cell populations. In animal models of influenza-related lung injury, HIF-1α-driven glycolysis facilitates the adaptation of tissue-resident alveolar macrophages to the hypoxic alveolar environment and maintains cell survival; enhancing this response may also reduce alveolar permeability and enhance host survival, suggesting a barrier-protective role in specific alveolar compartments (93). However, these results are primarily derived from tissue-resident alveolar macrophages and cannot be directly extrapolated to recruited monocyte-derived macrophages or all types of severe pneumonia. In contrast, in some recruited myeloid cells and persistent inflammatory environments, enhanced glycolysis supports cytokine production, migration, and other inflammatory effects; neutrophils rely mainly on glycolysis for chemotaxis, degranulation, and neutrophil extracellular trap formation (94, 95). Thus, the same metabolic pathway may mediate host defense, ensure cell survival, or drive inflammation in different immune cells, with functional shifts depending on local oxygen tension, stimulus intensity, and disease stage.

HIF-1α-related carbohydrate metabolism may also play an adaptive role in alveolar epithelial cells (96). Mechanical stretch models suggest that this response supports epithelial cell energy homeostasis and barrier integrity, reducing stretch-related injury (97). This further indicates that infection, hypoxia, and mechanical stress can directly shape the metabolic state of lung cells without requiring gut changes as a prerequisite. Therefore, HIF-1α-related glycolysis is better understood as a context-dependent cellular adaptation program, with its function depending on the specific cell, local microenvironment, and duration, rather than being uniformly classified as a pro-inflammatory or injury mechanism.

### 6.2 Lactate: circulating metabolic marker, receptor signaling, and epigenetic regulation

Enhanced glycolysis promotes the conversion of pyruvate to lactate. In severe pneumonia and other critical conditions, blood lactate levels are influenced by tissue hypoperfusion, adrenergic stress, cellular glycolysis, and hepatic/renal metabolism and clearance (98). Therefore, its clinical significance primarily reflects systemic perfusion and metabolic stress, rather than directly representing local alveolar lactate exposure or the metabolic status of specific immune cells. Circulating lactate is a whole-body composite metabolic indicator, while local alveolar lactate exposure and intracellular lactate metabolism operate at the tissue microenvironment and cellular levels, with differences in source, concentration, and functional significance.

Beyond being a metabolic byproduct, local lactate can act as an active signaling molecule via hydroxycarboxylic acid receptor 1 (HCAR1/GPR81), participating in metabolic communication among immune cells, epithelial cells, and endothelial cells. Experimental studies suggest that HCAR1/GPR81 signaling may modulate macrophage activation states, inhibit or reshape certain inflammatory responses, and influence angiogenesis, tissue repair, and barrier function (99). These effects are modulated by the interplay of lactate concentration, exposure time, local pH, receptor expression, and cell type, and thus cannot be categorically defined as pro-inflammatory or anti-inflammatory. Furthermore, local lactate may derive from the metabolism of resident inflammatory and structural cells or be affected by systemic circulation and tissue perfusion, necessitating a clear distinction from circulating lactate levels. Only after elucidating local exposure, target cell receptor responses, and subsequent tissue effects can the regulatory role of lactate across different levels be comprehensively assessed.

Lactate may also participate in epigenetic regulation through histone lactylation. This process can alter gene transcription programs in macrophages and other cells, potentially contributing to the transition from inflammation to repair phases. However, the function of histone lactylation is site-, cell type-, and disease stage-dependent, and cannot be simply equated with M2 macrophage polarization or anti-inflammatory responses; its cellular sources, temporal dynamics, and clinical significance in severe pneumonia patients remain insufficiently validated (100). Consequently, elevated circulating lactate, local alveolar lactate signaling, and intracellular histone lactylation constitute distinct biological phenomena across hierarchical levels; elucidating their interconnections requires careful interpretation of their sources, local exposure contexts, and specific cellular responses.

### 6.3 Mitochondrial damage, mtDNA release, and nucleic acid sensing signals

Mitochondrial dysfunction may be a key intracellular link connecting immunometabolic abnormalities to inflammatory responses. Hypoxia, inflammatory stimuli, oxidative stress, and mechanical stretch can disrupt mitochondrial membrane integrity, dynamic balance, and quality control, leading to restricted oxidative phosphorylation, reduced ATP production, and accumulation of mitochondrial reactive oxygen species (mtROS) (101). Moderate ROS levels contribute to pathogen clearance and cell signaling, while excessive accumulation can exacerbate oxidative damage (102). When mitochondrial membrane permeability increases or damaged mitochondria are inadequately cleared, mitochondrial DNA (mtDNA) can enter the cytosol or be released extracellularly, activating different nucleic acid sensing pathways as damage-associated molecular signals (103).

Cytosolic mtDNA can be recognized by cGAS, promoting cyclic GMP–AMP (cGAMP) production, which subsequently activates STING-TBK1-IRF3 signaling to induce type I interferon and interferon-stimulated gene expression. In contrast, extracellular mtDNA, after uptake into endosomes, may activate TLR9-related inflammatory signaling (104). Meanwhile, mtDNA release often coexists with increased mtROS, ion homeostasis disruption, and mitochondrial stress, promoting NLRP3 inflammasome activation, though mtDNA should not be considered its sole or direct trigger (105). These responses can participate in anti-pathogen defense but may also evolve into drivers of tissue damage under sustained or excessive activation, with functional outcomes depending on signal intensity, duration, and cellular context.

In the lungs, these signals may affect alveolar macrophages, alveolar epithelial cells, and vascular endothelial cells, contributing to type I interferon responses, sterile inflammation, barrier permeability changes, and tissue injury. Mechanical stretch and hyperoxia exposure may amplify this process by enhancing mitochondrial stress (106). However, cGAS-STING and type I interferon signaling can both participate in anti-pathogen defense and, under sustained or excessive activation, exacerbate inflammation, with effects depending on pathogen type, cell source, signal intensity, and disease stage (107). Furthermore, detecting mtDNA in circulation or tissues does not directly determine its origin from the gut, lungs, or other damaged tissues, and the relative contributions of different sources to systemic inflammation remain poorly defined. Circulating mtDNA cannot substitute for direct evidence of nucleic acid sensing pathway activation in pulmonary target cells. Current evidence primarily comes from *in vitro* studies and *in vivo* models of hyperoxia, ventilator-induced lung injury, and other ALI/ARDS models; the cellular sources, subcellular localization, dynamic changes, and relative contributions of different nucleic acid sensing pathways in severe pneumonia patients remain unclear. Therefore, mtDNA-related signals are better regarded as candidate mechanisms and research directions rather than established disease-specific intervention targets.

### 6.4 Gut metabolites and immune cell phenotype modulation

Gut microbiota-derived metabolites may influence host immune responses through receptor signaling, epigenetic regulation, and bone marrow-mediated remote immune programming, with evidence primarily from animal, cellular, and other disease studies. This process involves sequential steps including metabolite production, absorption, systemic exposure, and target cell response. SCFAs can signal through receptors such as GPR43/FFAR2, GPR41/FFAR3, and GPR109A/HCAR2, and also regulate gene transcription by inhibiting HDACs, thereby affecting the differentiation, activation, migration, and effector functions of regulatory T cells, macrophages, neutrophils, dendritic cells, and other T cell subsets.

Circulating SCFAs, in addition to directly acting on peripheral and pulmonary immune cells, may also modulate the proliferation, differentiation, and functional programming of bone marrow hematopoietic stem/progenitor cells and myeloid precursors, altering the output and response phenotype of monocytes, neutrophils, and dendritic cells, providing a potential mechanistic basis for gut metabolites influencing remote immune responses (108). However, this process requires multiple sequential steps—metabolite production, intestinal absorption, systemic transport, effective exposure in bone marrow or lungs, and target cell response—so changes in microbiota composition or single metabolite levels alone are insufficient to prove a complete gut-lung functional connection.

The impact of gut metabolites on the Treg/Th17 balance should not be simplified as unidirectional inhibition of Th17 responses. When moderately activated, IL-17A produced by Th17 and γδ T17 cells promotes neutrophil recruitment, supporting mucosal barrier defense and clearance of extracellular bacteria and fungi; however, when the response is sustained, overly intense, or inadequately regulated by Tregs, it may lead to excessive neutrophil recruitment, persistent inflammation, and tissue barrier damage (109). Thus, the net effect of IL-17A-related responses depends on pathogen type, cell source, response intensity, local microenvironment, and disease stage. Bile acids can influence macrophage activation, epithelial barrier, and energy metabolism via receptors such as FXR and TGR5 (110). Tryptophan-derived indole metabolites may participate in mucosal immunity and barrier protection through the AhR–IL-22 pathway (111). Therefore, microbiota metabolites may support host defense, inflammation limitation, and barrier maintenance, but may also promote inflammatory responses under specific pathogen backgrounds, exposure levels, or disease stages, with their direction of action inseparable from the specific biological context. However, current evidence regarding SCFAs-mediated bone marrow immune programming, as well as Treg/Th17 balance and IL-17A-related effects, remains primarily from animal, cellular, and other preclinical models. Longitudinal data simultaneously linking gut microbiota and metabolite changes, effective systemic exposure, immune cell responses, and pulmonary effects in severe pneumonia patients are still lacking. Thus, these studies currently support the functional feasibility of gut metabolites participating in remote immune regulation but are insufficient to establish a complete cross-organ evidence chain in humans.

Overall, existing evidence supports significant immunometabolic reprogramming in severe pneumonia, but its sources and functions are highly heterogeneous. Pulmonary infection, hypoxia, inflammation, mechanical stress, and therapeutic exposure can directly induce cellular metabolic changes, with effects manifesting as host defense, cell survival, and tissue repair depending on cell type, tissue compartment, and disease stage, or shifting toward inflammation and tissue damage under sustained or excessive stimulation. The regulation of this process by gut metabolites has preclinical mechanistic support, but the source, temporal sequence, effective exposure, and independent contribution in patients have not been established. Therefore, immunometabolism is currently better conceptualized as a dynamic cellular response layer shaped by multiple local and systemic inputs, rather than an established gut-lung cross-organ intermediary (**Figure 4**).

**Figure 4:**
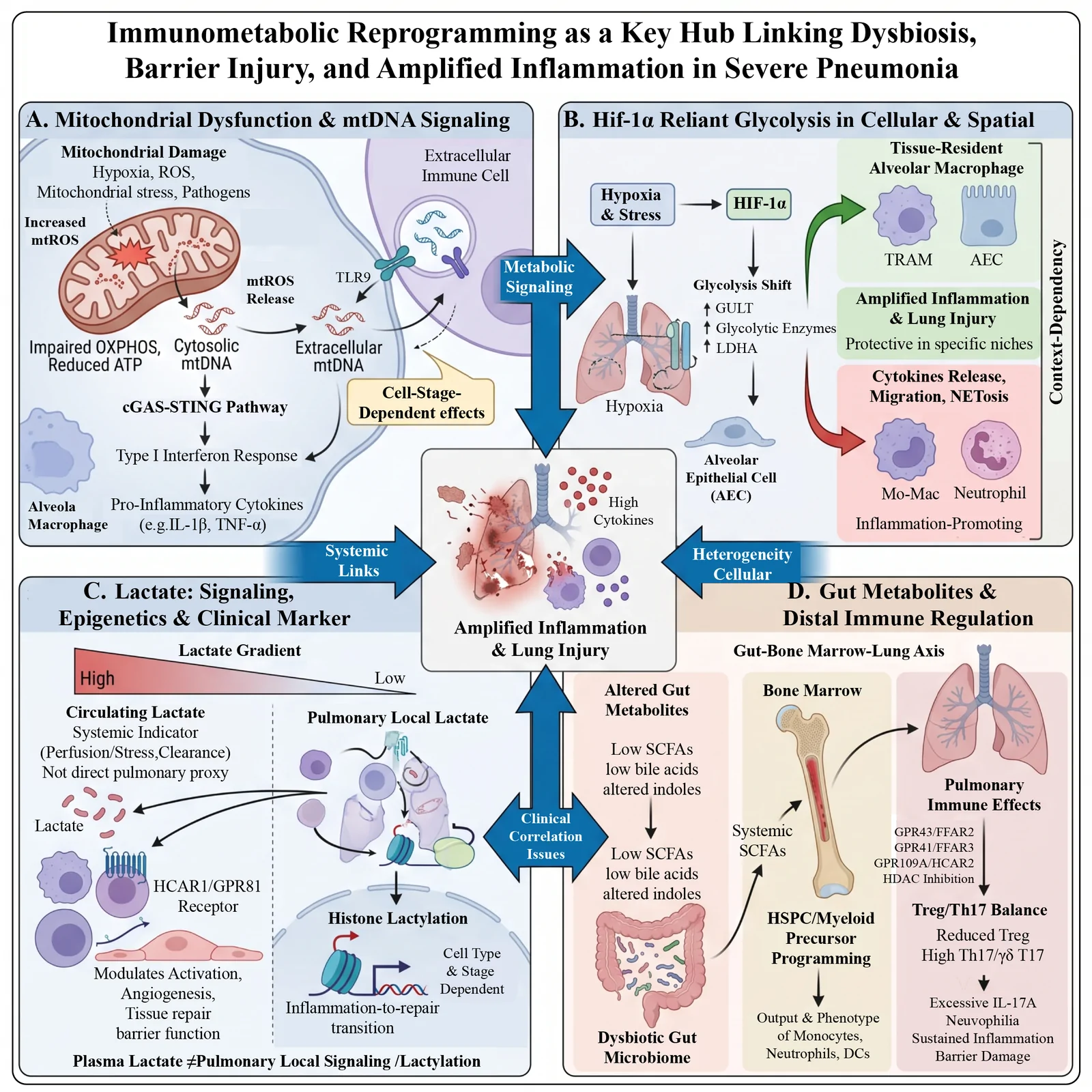
Immunometabolic reprogramming and candidate cross-organ connections in the multisystem response to severe pneumonia. This figure outlines how immunometabolic abnormalities amplify lung inflammation in severe pneumonia. **(A)** Hypoxia, ROS, pathogens, and mitochondrial stress lead to impaired OXPHOS, reduced ATP, increased mtROS, and mtDNA leakage, enhancing alveolar macrophage inflammation through TLR9, cGAS-STING, and inflammatory factor responses. **(B)** Hypoxia and stress activate HIF-1α-dependent glycolysis, promoting heterogeneous inflammatory responses in AECs, tissue-resident macrophages, monocytes/macrophages, and neutrophils. **(C)** Lactate serves both as a circulating perfusion/clearance indicator and, locally in the lung, regulates immune activation, angiogenesis, barrier function, and inflammation-repair transition through HCAR1/GPR81 signaling and histone lactylation. **(D)** Gut microbiota imbalance leads to reduced or altered SCFAs, bile acids, and indole metabolites, which can regulate myeloid cell output, Treg/Th17 balance, and lung inflammation via the bone marrow–lung axis. (Solid black arrows indicate relatively clear metabolic or inflammatory pathways; thick blue arrows indicate systemic connections between different modules and central lung injury, cell heterogeneity, or clinical relevance issues; green curved arrows indicate potential protective or regulatory effects in specific niches; red curved arrows indicate inflammatory amplification or pro-injury effects; dashed lines/dividers indicate different spatial regions, cell stages, or links with insufficient evidence. ↑/↓ indicate increase or decrease, respectively. AEC, alveolar epithelial cell; ATP, adenosine triphosphate; cGAS, cyclic GMP-AMP synthase; DCs, dendritic cells; FFAR2, free fatty acid receptor 2; FFAR3, free fatty acid receptor 3; GLUT, glucose transporter; GPR41, G protein-coupled receptor 41; GPR43, G protein-coupled receptor 43; GPR81, G protein-coupled receptor 81; GPR109A, G protein-coupled receptor 109A; HCAR1, hydroxycarboxylic acid receptor 1; HCAR2, hydroxycarboxylic acid receptor 2; HDAC, histone deacetylase; HIF-1α, hypoxia-inducible factor 1-alpha; HSPC, hematopoietic stem and progenitor cell; IL-1β, interleukin-1 beta; IL-17A, interleukin-17A; LDHA, lactate dehydrogenase A; Mo-Mac, monocyte-derived macrophage; mtDNA, mitochondrial DNA; mtROS, mitochondrial reactive oxygen species; NETosis, neutrophil extracellular trap formation; OXPHOS, oxidative phosphorylation; ROS, reactive oxygen species; SCFAs, short-chain fatty acids; STING, stimulator of interferon genes; Th17, T helper 17 cell; TLR9, Toll-like receptor 9; TNF-α, tumor necrosis factor-alpha; TRAM, tissue-resident alveolar macrophage; Treg, regulatory T cell).

## 7 Mechanical ventilation and related therapeutic exposures: dynamic regulatory factors in critically Ill hosts

In the critically ill host environment of severe pneumonia, mechanical ventilation has relatively well-established direct pulmonary mechanical effects, while coexisting with multiple exposures such as sedation, immobilization, hemodynamic changes, oxygenation and ventilation status, antibiotics, and nutritional support. Therefore, this chapter first distinguishes the direct effects of mechanical ventilation on VILI from the systemic effects of the accompanying therapeutic environment, and further examines the temporal and functional relationships between these factors and intestinal and immune-metabolic changes.

### 7.1 Mechanical stress and pulmonary inflammatory spillover

Mechanical ventilation is an important life support measure for maintaining oxygenation and ventilation in patients with severe pneumonia and respiratory failure, but inappropriate ventilation intensity may exacerbate primary lung injury (112). Excessive tidal volume, driving pressure, plateau pressure, or mechanical power can cause alveolar overdistension, repeated collapse-recruitment, and shear stress injury, thereby inducing or aggravating VILI (113). In addition to structural damage, VILI can also cause mechanical stress-related biotrauma. Damaged alveolar epithelial and vascular endothelial cells can release pulmonary-derived DAMPs and inflammatory mediators, facilitating the translocation of local inflammation into the systemic circulation and potentially increasing the inflammatory burden on distant organs (114).

The direct mechanical injury to lung tissue from mechanical ventilation and its inflammatory effects have a relatively clear evidence base, but the evidence for further causing intestinal abnormalities is significantly weaker. Some animal experiments and observational studies in critically ill patients suggest that higher ventilation intensity, more severe lung injury, and systemic inflammatory response may be associated with increased intestinal permeability, elevated circulating LPS levels, or multi-organ dysfunction (115). However, these changes are frequently confounded by shock, perfusion abnormalities, and other therapeutic exposures. Therefore, there is strong direct evidence that mechanical ventilation causes or exacerbates VILI, but whether it independently induces changes in the intestinal barrier or microbiome remains challenging to disentangle from disease severity, circulatory disturbances, and concomitant therapeutic factors. Current research characterizes these changes as concomitant phenomena in the critically ill environment, but a stable “mechanical ventilation → intestinal injury” causal continuum has not yet been established.

### 7.2 Mechanical ventilation, sedation, and intestinal dysfunction

Many patients with severe pneumonia receiving invasive mechanical ventilation require varying degrees of sedation and analgesic support. Sedative and analgesic drugs, immobilization, and positive pressure ventilation itself can all affect gastrointestinal function (22). Opioids can inhibit the excitability of the enteric nervous system through intestinal μ receptors, delaying gastrointestinal motility; some sedatives may also affect gastrointestinal motility through central and autonomic nervous system regulation (116). Decreased gastrointestinal motility can lead to intestinal content stasis, reduced tolerance to enteral nutrition, and enrichment of opportunistic pathogens, thereby increasing the likelihood of microbiome dysbiosis and barrier dysfunction (117). Furthermore, positive pressure ventilation can indirectly alter splanchnic perfusion by increasing intrathoracic pressure and affecting venous return and cardiac output (118). For patients with severe pneumonia complicated by shock, hypoxia, or hemodynamic instability, intestinal hypoperfusion and reperfusion injury may induce intestinal epithelial cell apoptosis, abnormal claudin expression, and increased intestinal permeability. Delayed initiation of enteral nutrition, deep sedation, prolonged immobilization, and broad-spectrum antibiotic exposure may also simultaneously affect gastrointestinal motility, microbiota composition, and barrier function (119). These factors often change synchronously with the disease course, but their mechanisms of action differ: positive pressure ventilation and circulatory status primarily modulate splanchnic perfusion, sedation and immobilization govern gastrointestinal motility, nutritional changes determine the supply of substrates to the intestinal epithelium, and antibiotics directly alter the selective pressure on the microbiome. Therefore, sedation, immobilization, circulatory status, nutrition, and drug exposure should be evaluated as independent and time-varying clinical factors, rather than being subsumed under the single variable of “mechanical ventilation.”

### 7.3 Potential impact of changes in oxygenation and ventilation status on immune metabolism

Changes in oxygenation and ventilation status during mechanical ventilation may affect host immune metabolism. Hypoxia-induced HIF-1α-related metabolic adaptation is not a uniformly harmful response; its effects depend on cell type, tissue compartment, and duration of stimulation. In tissue-resident alveolar macrophages and alveolar epithelial cells, this response may support hypoxic adaptation, cell survival, and barrier maintenance; whereas in some recruited inflammatory cells or under persistent stimulation, it may enhance inflammatory effects (120). Therefore, hypoxia-related metabolic reprogramming may support cell survival, barrier function, and host defense adaptation, but may also enhance inflammatory effects in specific cell types or under conditions of persistent stimulation.

Exposure to high oxygen concentrations can lead to redox imbalance, mitochondrial stress, and damage to alveolar epithelial and endothelial cells (121, 122). Its effects are also dose- and time-dependent, and are co-regulated by the degree of underlying lung injury, antioxidant capacity, and ventilation strategy. Carbon dioxide retention and changes in acid-base status can also affect inflammatory and metabolic responses. Permissive hypercapnia is a physiological state that may occur during lung-protective ventilation, but its effects depend on pH, exposure time, cell type, and disease stage (123, 124). Therefore, the metabolic changes accompanying hypoxia, hyperoxia, and hypercapnia may reflect physiological adaptation, but can also progress to injurious stress when exposure is too intense or prolonged. The above mechanisms are primarily derived from *in vitro* studies, preclinical animal models, and non-pneumonia ARDS models.

In patients with severe pneumonia, hypoxia, hyperoxia, hypercapnia, and mechanical stress often coexist and are jointly influenced by disease severity, hemodynamic status, and ventilation strategy. These factors can collectively act on cellular energy metabolism, redox homeostasis, mitochondrial function, and inflammatory signaling, making the immune-metabolic phenotype highly dynamic. Therefore, the significance of oxygenation and ventilation-related metabolic changes should be holistically evaluated based on pathogen load, tissue demand, exposure intensity, and organ function, and cannot be reduced to a single HIF-1α, lactate, or mitochondrial pathway.

### 7.4 Direct benefits and potential distant effects of lung-protective ventilation

The primary clinical significance of lung-protective ventilation is to reduce alveolar overdistension, repeated collapse-recruitment, and mechanical power-related injury, thereby lowering the risk of VILI. Its clinical application is based on independent evidence from ARDS and mechanical ventilation management, rather than on a proven direct regulatory effect on the gut microbiota or intestinal barrier (125). By limiting tidal volume, plateau pressure, and driving pressure, and by the optimal titration of PEEP, lung-protective ventilation can reduce pulmonary mechanical stress, DAMP release, and inflammatory mediator spillover (126). Theoretically, reducing the pulmonary inflammatory load may decrease distant organ stress, but this effect is not equivalent to a direct improvement in the intestinal microbiome or barrier.

PEEP and ventilation intensity can also affect splanchnic perfusion through hemodynamic changes. Excessively high intrathoracic pressure may reduce venous return and cardiac output, increasing the risk of intestinal hypoperfusion; while insufficient ventilation or repeated alveolar collapse-recruitment may exacerbate hypoxia and pulmonary inflammation (127, 128). Therefore, lung-protective ventilation does not provide unidirectional protection for the intestinal milieu; rather, it requires individualized balancing between lung recruitment, oxygenation improvement, mechanical stress control, and hemodynamic stability. The same ventilation strategy may have different directional effects on pulmonary mechanical load and distant organ perfusion. Consequently, its overall impact, which depends on lung mechanics, circulatory reserve, and disease stage, cannot be simply inferred as unidirectional protection for the intestine.

Overall, current evidence clearly supports a direct functional link between mechanical ventilation intensity and VILI, and demonstrates that positive pressure ventilation, oxygenation status, and hemodynamic changes can affect the systemic physiological environment. In contrast, human evidence linking mechanical ventilation to changes in the gut microbiota, barrier, and immune metabolism is primarily derived from observational associations, and is influenced by factors such as sedation, nutrition, antibiotics, shock, and the severity of the primary disease. The temporal sequence and independent cross-organ effects have not yet been established. Therefore, mechanical ventilation is better understood as a therapeutic environment with clear direct pulmonary effects, accompanied by multiple systemic exposures, while current evidence is insufficient to determine an independent mechanical ventilation-related lung-gut feedback mechanism (**Figure 5**).

**Figure 5:**
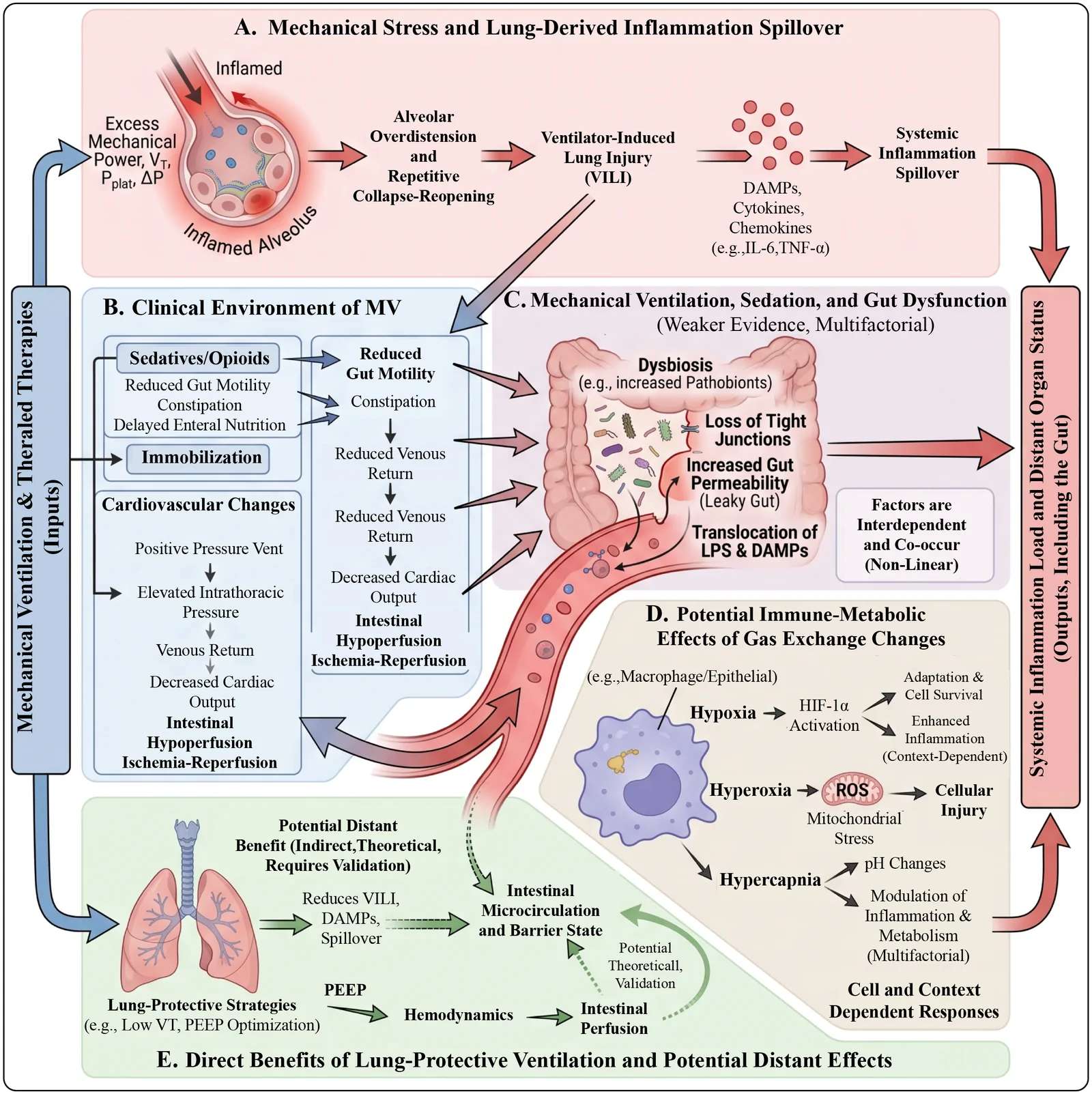
Mechanistic framework of mechanical ventilation-related lung–gut interaction and systemic inflammation amplification. This figure outlines the multi-layered effects of mechanical ventilation on the lungs, gut, and systemic inflammation in severe pneumonia/ARDS. **(A)** Excessive mechanical power, tidal volume, plateau pressure, or driving pressure may induce alveolar overdistension and recurrent collapse and recruitment, inducing VILI and promoting systemic inflammation through the spillover of DAMPs, cytokines, and chemokines. **(B, C)** Sedatives and opioids, immobilization, delayed enteral nutrition, and hemodynamic changes related to positive pressure ventilation can collectively reduce intestinal motility and perfusion, thereby fostering microbiome imbalance, loss of tight junctions, increased intestinal permeability, and translocation of LPS/DAMPs. **(D)** Hypoxia, hyperoxia, and hypercapnia can modulate immune cell and epithelial cell responses through HIF-1α, ROS, pH changes, and metabolic and inflammatory regulatory pathways. **(E)** Lung-protective ventilation may produce distant protective effects by reducing VILI and inflammatory spillover, and by improving hemodynamics and intestinal perfusion, although these mechanisms require further validation. (Solid black arrows indicate relatively clear clinical or pathological processes; thick red arrows indicate injury progression, inflammatory spillover, or systemic spread; blue arrows indicate mechanical ventilation and therapeutic inputs; solid green arrows indicate potential beneficial effects of lung-protective ventilation; dashed green arrows indicate theoretical or yet-to-be-validated distant protective relationships; parallel arrows indicate the concerted action of multiple factors, not a single pathway; circular/curved arrows indicate feedback amplification. MV, mechanical ventilation; VT, tidal volume; Pplat, plateau pressure; ΔP, driving pressure; VILI, ventilator-induced lung injury; DAMPs, damage-associated molecular patterns; IL-6, interleukin-6; TNF-α, tumor necrosis factor-alpha; LPS, lipopolysaccharide; HIF-1α, hypoxia-inducible factor 1-alpha; ROS, reactive oxygen species; PEEP, positive end-expiratory pressure).

## 8 Gut–lung–immune metabolic abnormalities and clinical outcomes: associations, prediction, and causal interpretation

The clinical outcomes of patients with severe pneumonia are determined by the interplay of the primary infection and lung injury, systemic inflammation, shock and organ dysfunction, host reserve, and therapeutic exposures such as mechanical ventilation, antibiotics, sedation, and nutrition. In this context, abnormalities in the gut microbiota, barrier, and immune metabolism may either accompany changes in disease severity or provide additional risk prediction or mechanistic information. Their clinical significance depends not only on their association with outcomes such as ARDS, VAP, MODS, or mortality, but also on their ability to provide independent or incremental information beyond traditional metrics of disease severity, circulatory status, organ function, and therapeutic exposures. Furthermore, it relies on whether the relevant biological changes demonstrate consistent temporal and functional correlations with patient outcome trajectories. Therefore, this chapter focuses on distinguishing disease-associated changes, candidate risk markers, and potentially functional mechanistic factors, and evaluates the extent to which current evidence supports these distinctions.

### 8.1 ARDS progression and oxygenation deterioration

ARDS progression and oxygenation deterioration are important clinical manifestations of lung injury associated with severe pneumonia. Existing human studies have mainly observed that systemic inflammation, metabolic stress, and candidate markers of intestinal barrier injury such as D-lactate, DAO, and I-FABP co-occur with decreased PaO_2_/FiO_2_ ratio, lung injury severity, need for mechanical ventilation, and poor prognosis (129, 130). However, the relevant evidence mostly comes from general ARDS, sepsis, or mixed critical illness cohorts, and longitudinal studies that simultaneously and continuously monitor intestinal barrier status and oxygenation changes in patients with severe pneumonia remain limited.

Most of the above studies are observational and are confounded by factors such as primary etiology, source of infection, shock, hemodynamic status, organ dysfunction, and exposure to mechanical ventilation and medications. Therefore, it is difficult to determine the temporal sequence and independent association between elevated intestinal barrier markers and oxygenation deterioration. Elevated D-lactate, DAO, and I-FABP can also accompany systemic hypoperfusion, shock, and multi-organ stress, and thus may partially reflect overall disease severity rather than specifically indicating a lung injury process mediated by intestinal barrier abnormalities (90). Heterogeneous lung tissue can cause local overdistension and repeated collapse–recruitment, which can superimpose VILI on primary pneumonia injury (131, 132). Consequently, there is currently insufficient robust evidence to determine whether these intestinal barrier markers improve predictive performance for ARDS progression or oxygenation deterioration beyond established severity indicators such as SOFA/APACHE scores, circulatory status, and pulmonary mechanics parameters.

Preclinical studies suggest that microbial-associated and tissue damage signals entering the blood or lymphatic system after intestinal barrier damage, under conditions where they escape hepatic and systemic clearance, maintain biological activity, and reach target cells in the lungs, may affect the pulmonary vascular endothelium, alveolar macrophages, and the alveolar–capillary barrier, thus providing biological plausibility for gut-derived signals participating in lung injury (133). However, detecting related molecules in the circulation does not prove their intestinal origin, nor does it prove that they have escaped hepatic clearance, entered the local lung compartment, or reached concentrations sufficient to activate pulmonary target cells (134). Furthermore, the initial injury site, degree of hypoperfusion, and pathological sequence in the gut injury-first model are inconsistent with those in severe pneumonia. Therefore, current human evidence supports a clinical association between intestinal barrier injury markers and ARDS severity and oxygenation deterioration, but their independent predictive value has not yet been established; preclinical studies provide mechanistic feasibility for gut-derived signals affecting lung injury, but have not yet established continuous functional evidence at the patient level. Currently, these markers are more suitable as candidate risk or mechanistic markers rather than established independent drivers of ARDS progression.

### 8.2 Increased risk of VAP and secondary infections

Patients with severe pneumonia often receive broad-spectrum antibiotics, mechanical ventilation, and sedation and analgesia during ICU treatment, and frequently present with concomitant factors such as artificial airways, aspiration, malnutrition, and impaired host immune function. These disease- and treatment-related factors can collectively reduce microbial diversity, promote the enrichment of opportunistic and drug-resistant pathogens, and weaken colonization resistance (135, 136). Changes in the microbiota of the oropharynx, gastrointestinal tract, and lower respiratory tract may therefore be associated with the occurrence of VAP and other secondary infections (137). Synchronous changes in the microbiota of different compartments may reflect common antibiotic and host selection pressures, or may, in some patients, suggest transmission links between reservoirs, with different clinical implications.

General ICU and VAP studies suggest that antibiotic selection pressure can shift the pathogen spectrum towards Acinetobacter baumannii, Pseudomonas aeruginosa, and drug-resistant Enterobacteriaceae, thereby complicating therapeutic management (138, 139). Related studies also suggest that Th17 and γδ T17 cell-associated IL-17A responses can promote mucosal neutrophil recruitment and clearance of extracellular pathogens, and thus may participate in defense against VAP and other secondary infections (140). However, when IL-17A responses are sustained, excessive, or lack Treg regulation, they may also promote excessive neutrophil recruitment, persistent lung inflammation, and barrier damage (141). Therefore, both microbial changes and immune responses are highly context-dependent, and their clinical effects cannot be explained by a single microbial characteristic or a single immune pathway. Overall, VAP risk is more likely to be multifactorially determined by artificial airways, antibiotic selection pressure, aspiration, disease severity, host immune status, and the microbiota of different compartments. Current evidence supports an association between microbial remodeling and secondary infections, and provides clues to strain sources in some patients, but is insufficient to prove that gut pathogen translocation has incremental predictive value independent of traditional VAP risk factors or a universal pathogenic role.

### 8.3 MODS, ICU-acquired weakness, and long-term prognosis

Systemic inflammation, coagulation activation, microcirculatory disturbances, and metabolic abnormalities may all be associated with extrapulmonary organ dysfunction. Studies involving patients with sepsis, trauma, and other critical illnesses suggest that gut-derived inflammatory signals, spillover of lung inflammation, and microcirculatory disturbances may jointly contribute to impaired liver, kidney, circulatory, and neurological function (142, 143). In patients with severe pneumonia, MODS is closely associated with higher mortality, longer mechanical ventilation duration, and longer ICU stay (144). Intestinal barrier and immune metabolic abnormalities may change with organ function deterioration, but whether they can independently explain MODS risk beyond shock, infectious burden, hypoxia, and microcirculatory disturbances has not been fully demonstrated.

Persistent inflammation, inadequate nutritional intake, increased protein breakdown, mitochondrial dysfunction, and prolonged immobilization are also associated with ICU-acquired weakness. Low albumin, malnutrition, and metabolic abnormalities often coexist with difficulty in weaning, prolonged hospital stay, and poor functional recovery (145). The gut microbiota and immune metabolic status may participate in this complex recovery process, but there is currently insufficient evidence to determine their independent predictive value and mediating role (146). For long-term outcomes such as pulmonary fibrosis, cognitive dysfunction, cardiac events, readmission, and long-term survival, existing research is even more limited, and related associations may also be influenced by age, underlying diseases, degree of organ damage, ICU exposure, and recovery processes (147). Therefore, at this stage, the value of gut microbiota, barrier, and immune metabolic markers in MODS and long-term prognosis is closer to candidate severity or risk stratification information rather than established independent pathogenic factors. Their true incremental clinical value needs to be further determined through longitudinal models, incremental predictive analysis, and external validation based on traditional severity scores and major therapeutic exposures.

Overall, existing clinical studies have suggested that gut microbiota, barrier, and immune metabolic markers are variably associated with ARDS progression, secondary infections, MODS, and long-term recovery, but this association itself is insufficient to prove their independent clinical significance. The most critical issue at present is whether these markers can provide stable incremental predictive information beyond traditional factors such as infectious burden, lung injury severity, shock, organ function, and ICU therapeutic exposures, and whether their dynamic changes precede and are functionally linked to changes in patient outcomes. At this stage, most markers are more suitable as candidate risk or severity markers; only after demonstrating efficacy through functional intervention, alongside evidence of temporality and independent prediction, can their clinical value as cross-organ mechanistic markers or therapeutic targets be further defined (**Table 1**).

**Table 1:** Evidence evaluation of gut–lung–immune metabolic abnormalities and clinical outcomes in severe pneumonia.

Clinical outcome	Candidate association	Evidence category	Key evidence	Evidence maturity	Current clinical positioning	Reference
ARDS progression and oxygenation deterioration	Gut barrier injury, systemic inflammation, and candidate gut-derived signals.	Cross-departmental association Among them, “enterogenous signal → lung injury” belongs to the candidate causal relationship.	Elevated D-lactic acid, DAO, and I-FABP are associated with decreased PaO_2_/FiO_2_, mechanical ventilation, and poor prognosis; signal sources, lung exposure, and causal sequence remain unconfirmed.	Low– moderate	Candidate severity/prognosis markers, not yet specific indicators of gut-derived lung injury.	(129–134)
VAP and secondary infections	Decreased flora diversity, enrichment of drug-resistant bacteria, and Th17/Treg imbalance.	Cross-departmental association “Intestinal pathogen migration → pulmonary infection” belongs to the candidate causal relationship.	Microecological disruption correlates with infection risk; IL-17A may both promote pathogen clearance and exacerbate lung inflammation.	Low– moderate	Useful for assessing infection risk and pathogen ecology, but unable to confirm gut pathogen translocation.	(135–141)
MODS	Inflammatory spillover, microcirculatory disturbance, coagulation, and metabolic abnormalities.	Cross- departmental association	Relevant indicators are associated with multi-organ injury and poor short-term outcomes, with evidence derived primarily from sepsis or mixed critical care cohorts.	Low	Exploratory multi-organ risk framework, not yet recognized as an independent pathogenic mechanism.	(142–144)
ICU-acquired weakness and long-term outcomes	Persistent inflammation, malnutrition, protein catabolism, mitochondrial and microecological abnormalities.	Cross- departmental association	These factors are associated with difficult weaning, impaired functional recovery, and prolonged hospitalization; however, evidence regarding long-term functional status, readmission rates, and survival remains limited.	Low/ exploratory	While this may serve as a candidate dimension for comprehensive risk assessment, it is currently insufficient to guide independent diagnostic or therapeutic decisions.	(145–147)

ARDS, acute respiratory distress syndrome; DAO, diamine oxidase; I-FABP, intestinal fatty acid-binding protein; MODS, multiple organ dysfunction syndrome; VAP, ventilator-associated pneumonia.

## 9 Potential intervention strategies: from basic ICU management to exploratory targeted therapy

Severe pneumonia-associated lung injury involves multiple intervenable levels, including pulmonary mechanical stress, circulatory and metabolic changes, gut microbiome and barrier function, and host immune responses. The clinical role of various strategies depends on the maturity of their respective evidence. Lung-protective ventilation, appropriate PEEP, early weaning, optimized sedation, and early enteral nutrition are basic supportive measures with high or relatively high clinical maturity and should be integral components of comprehensive management of severe pneumonia. Strategies such as probiotics, synbiotics, ω-3 fatty acids, and immunonutrition have certain theoretical foundations and partial clinical signals, but results remain inconsistent. Fecal microbiota transplantation (FMT) and targeted strategies involving NLRP3, TLR4, and intestinal alkaline phosphatase (IAP) remain in exploratory or early translational stages and should not be recommended as current standard treatments. These interventions can act on different components of the gut–lung–immune–metabolic framework, but their mechanisms of benefit and target populations need to be evaluated separately and should not be viewed as a therapeutic system targeting a single unified pathway.

### 9.1 Basic supportive measures: lung-protective ventilation, early weaning, and enteral nutrition

Lung-protective ventilation is a basic intervention with relatively mature evidence. Limiting tidal volume, plateau pressure, and driving pressure, setting PEEP appropriately, reducing mechanical power, and minimizing patient–ventilator asynchrony and unnecessary deep sedation help mitigate VILI and its induced systemic inflammatory spillover (148, 149). Prone positioning and ECMO can serve as important supportive measures for patients with severe or very severe ARDS, helping improve oxygenation, reduce pulmonary mechanical stress, or achieve “lung rest,” but their remote effects on gut microbiome, barrier function, or immune–metabolic status still lack sufficient clinical evidence (150, 151). Therefore, the main benefits of mechanical ventilation optimization lie in reducing VILI, decreasing pulmonary-derived inflammatory burden, and shortening unnecessary mechanical ventilation exposure, potentially indirectly alleviating distant organ stress under conditions of maintained hemodynamic stability.

Early weaning, optimized sedation, and reduced prolonged immobilization are equally important. Deep sedation, neuromuscular blockade, and prolonged mechanical ventilation exposure can affect gastrointestinal motility, enteral nutrition tolerance, and splanchnic perfusion, and may precipitate gut microbiome dysbiosis and barrier dysfunction (152). Therefore, while ensuring oxygenation and patient–ventilator synchrony, early assessment of spontaneous breathing, reducing unnecessary deep sedation, and shortening mechanical ventilation duration not only help reduce ventilator-associated complications but may also lower treatment-related burden on extrapulmonary organs by improving activity levels, gastrointestinal motility, and circulatory status. The potential gut-related benefits should be regarded as secondary benefits of comprehensive ICU management rather than the primary therapeutic rationale for these measures.

Early enteral nutrition is an important basic measure for maintaining intestinal barrier and nutritional metabolism. For patients with severe pneumonia who are relatively hemodynamically stable, early initiation of enteral nutrition helps preserve intestinal mucosal structure, support enterocyte metabolism, improve nutritional status, and potentially reduce spillover of gut-derived inflammatory signals (153, 154). However, early enteral nutrition does not equate to overfeeding; its implementation should be individualized based on shock status, vasopressor dosage, gastrointestinal tolerance, and aspiration risk. Therefore, its clinical value is primarily reflected in meeting nutritional requirements of critically ill patients and maintaining gastrointestinal function, while microbiome-related or cross-organ immune effects represent potential additional mechanisms worthy of further validation.

### 9.2 Microecology and nutritional intervention: high potential but inconsistent evidence

Probiotics, prebiotics, synbiotics, and postbiotics can theoretically improve microecological and intestinal barrier abnormalities in critical conditions by regulating gut microbiota, enhancing barrier function, increasing SCFAs production, and inhibiting pathogen colonization. Mechanistically, increased SCFAs production may also influence systemic immunity through receptor signaling, HDAC inhibition, and bone marrow myeloid cell programming, but these mechanisms are primarily supported by preclinical studies, and whether they further translate into pulmonary effects and clinical benefits remains unclear (155). Some clinical studies suggest that specific probiotic strains may reduce diarrhea, plasma LPS levels, or the incidence of VAP. However, existing studies exhibit significant heterogeneity in strain or formulation composition, dosage, treatment duration, intervention timing, and patient characteristics, leading to inconsistent results (156). Therefore, probiotics and synbiotics should not be regarded as ‘universally effective’; instead, their application must prioritize strain specificity, population dependency, and safety assessment. Crucially, a distinction must be made between improving gut microecology or biomarkers and improving clinical outcomes such as ARDS, VAP, mechanical ventilation duration, or mortality; these endpoints are not synonymous.

Omega-3 fatty acids and other immunonutrition strategies may play a role by modulating arachidonic acid metabolism, lipid inflammatory mediator production, macrophage polarization, and immunometabolic status (157, 158), but consistent evidence supporting their ability to improve clinical outcomes for severe pneumonia or ARDS is lacking, and thus they should not be recommended as definitive measures. Although glutamine serves as a vital energy substrate for intestinal epithelial cells and demonstrates barrier-protective effects in animal models, clinical studies in ICU patients have yielded inconsistent results; consequently, routine high-dose administration is not recommended (159). Therefore, at this stage, microecological and immunonutrition interventions are more suitable as individualized supportive strategies based on nutritional risk, gastrointestinal tolerance, infection risk, and metabolic status, rather than as substitutes for standard intensive care and lung-protective treatment. Their potential value should be validated separately according to specific agents, mechanisms of action, and patient phenotypes.

### 9.3 FMT and targeted therapy: still in the exploratory and early translational stage

FMT has been well-established in recurrent Clostridioides difficile infection, but its application in ICU patients with severe pneumonia or ARDS remains highly exploratory (160). Critically ill patients often have immune dysregulation, intestinal barrier disruption, ileus, antibiotic exposure, and increased infection risk, and FMT may pose risks of bacteremia, opportunistic infections, and donor-related complications (161). Therefore, FMT is currently more suitable as a small-scale exploratory study under strict ethical review or as a preclinical research direction, rather than as a routine intervention. Future evaluations should focus not only on microbiota reconstruction but also on pathogen transmission, resistance gene transfer, intestinal barrier integrity, and safety at different disease stages.

Therapies targeting TLR4/LBP, HMGB1, NLRP3 inflammasome, and IAP offer candidate intervention directions from different levels, such as inflammatory signal regulation and barrier protection. TLR4 inhibition, anti-HMGB1 strategies, NLRP3 small-molecule inhibitors, and IAP-mediated LPS detoxification have demonstrated promise in animal experiments or early studies (162). However, these strategies are currently mostly in the preclinical or early translational stage, lacking sufficient efficacy and safety evidence in patients with severe pneumonia. Previous clinical trials targeting single anti-inflammatory pathways have not consistently yielded ideal results in critical care, also suggesting that patient heterogeneity, timing of administration, disease stage, and combination therapy strategies may determine their translational value (163). Moreover, the same inflammatory pathway may be involved in both host defense and tissue damage; thus, the fact that a pathway can be pharmacologically modulated does not mean it constitutes an appropriate therapeutic target in all patients with severe pneumonia.

Therefore, targeted strategies such as NLRP3, TLR4, and IAP should be considered as future directions for precision intervention, rather than being presented as existing mature treatment regimens. Overall, at this stage, the core should remain basic ICU management, including lung-protective ventilation, optimized sedation, early weaning, and rational enteral nutrition; microecological, immunonutrition, and molecular targeted strategies should be independently validated based on specific biological abnormalities, evidence maturity, and safety. Whether they produce additional clinical benefits through lung-gut cross-organ interactions remains to be further studied (**Table 2**).

**Table 2:** Potential intervention strategies and evidence maturity for the gut-lung-immune-metabolic axis in severe pneumonia-associated lung injury.

Intervention strategy	Primary site of action	Evidence maturity	Current clinical positioning	Key limitations	Reference
Lung-protective ventilation and appropriate PEEP	Reduce tidal volume, plateau pressure, driving pressure, and mechanical power, decreasing VILI and inflammatory spillover.	High	Standard ICU basic treatment.	Requires individualized balancing of lung recruitment, oxygenation, and hemodynamics.	(148, 149)
Prone positioning ventilation and ECMO	Improve oxygenation in severe ARDS, reduce local mechanical stress, or achieve “lung rest.”	Medium– high	Rescue supportive measures for severe or extremely severe ARDS.	Direct regulatory effects on the gut–lung–immune–metabolic axis remain unconfirmed.	(150, 151)
Sedation optimization and early weaning	Reduce deep sedation, immobilization, and mechanical ventilation exposure, improving gastrointestinal motility and enteral nutrition tolerance.	Medium– high	Important component of standard supportive management.	Limited by oxygenation, patient–ventilator synchrony, and respiratory muscle load.	(152)
Early enteral nutrition	Maintain intestinal mucosal structure and epithelial metabolism, improving nutritional status and barrier function.	Medium– high	Basic nutritional strategy for hemodynamically stable patients.	Avoid overfeeding and assess shock, aspiration, and gastrointestinal tolerance.	(153, 154)
Probiotics, prebiotics, synbiotics, and postbiotics	Modulate microbiota, enhance colonization resistance and barrier function, and promote SCFA production.	Low– medium	Selective adjunctive strategy, not yet routine recommendation.	Significant heterogeneity in strains, doses, treatment duration, and populations; safety in critically ill patients requires evaluation.	(155, 156)
ω-3 fatty acids and immunonutrition	Modulate lipid inflammatory mediators, macrophage function, and immunometabolism.	Low– medium	Individualized nutritional support, not recommended as a definitive gut–lung axis therapy.	Inconsistent benefits regarding clinical outcomes; target populations and optimal formulations remain undefined.	(157, 158)
Glutamine supplementation	Provide metabolic substrates for intestinal epithelial cells, potentially supporting barrier function.	Low	Routine high-dose use not recommended.	ICU study results and potential safety risks.	(159)
FMT	Rapidly restore intestinal microecology and microbial function.	Exploratory	Limited to preclinical studies or early trials under strict regulation.	High risks associated with bacteremia and opportunistic infections, as well as challenges related to donor screening and administration routes.	(160, 161)
TLR4/LBP, HMGB1, NLRP3, and IAP targeted therapy	Inhibit LPS-related inflammatory amplification and inflammasome activation, and protect the intestinal barrier.	Preclinical/ early translational	Future direction for precise biomarker-stratified intervention.	Lack of evidence on efficacy, safety, and optimal therapeutic window in severe pneumonia populations.	(162, 163)

Evidence maturity: “High” indicates stable clinical evidence and inclusion in routine critical care supportive management; “Medium-high” indicates relatively clear clinical value but limited evidence for specific effects on the gut-lung axis; “Low-medium” indicates partial clinical signals but with study heterogeneity or inconsistent results; “Preclinical/exploratory” indicates evidence mainly from animal experiments, mechanistic studies, or other disease populations. ARDS, acute respiratory distress syndrome; ECMO, extracorporeal membrane oxygenation; FMT, fecal microbiota transplantation; IAP, intestinal alkaline phosphatase; LBP, lipopolysaccharide-binding protein; PEEP, positive end-expiratory pressure; SCFAs, short-chain fatty acids; VILI, ventilator-induced lung injury.

## 10 From association to functional validation: Future research directions for gut–lung–immune–metabolic cross-organ communication in severe pneumonia

### 10.1 Establishing a multi-compartment longitudinal dynamic cohort for patients with severe pneumonia

Future efforts should focus on establishing prospective, longitudinal dynamic cohorts. Sequential collection of fecal, airway, and blood samples should be conducted at representative time points, including early ICU admission, key mechanical ventilation milestones, stages of infection control, and pre- and post-weaning periods. Concurrently, data on intestinal and airway microbiota, intestinal barrier markers, and inflammatory and metabolic profiles should be recorded alongside clinical outcomes, including PaO_2_/FiO_2_ ratios, ARDS severity, VAP incidence, duration of mechanical ventilation, ICU length of stay, and mortality. The research focus should not be limited to describing “microbial differences” at different time points but should compare several potentially coexisting explanations: whether microbial changes primarily reflect disease severity and the shared host response; whether they are secondary to pulmonary infection, shock, or ICU treatment; or whether, in some patients, they precede subsequent pulmonary deterioration and carry independent predictive or functional significance. Additionally, it should be evaluated whether there are reproducible strain-level source connections between the gut and airway microbiota, or whether the changes primarily manifest as a common remodeling driven by antibiotics, nutrition, and the critical host environment. Through time-series and multi-compartment joint analysis, parallel changes, intestinal alterations secondary to pulmonary/systemic responses, and candidate feedback loops with potential downstream pulmonary effects can be further distinguished.

### 10.2 Integrating multi-omics, source tracing, and functional validation

Metagenomics, metabolomics, transcriptomics, proteomics, as well as single-cell and spatial omics, can depict the microbiota and host response in severe pneumonia from different levels. Metagenomics can resolve the composition and functional potential of gut and airway microbiota; metabolomics can detect SCFAs, bile acids, tryptophan metabolites, lactate, and lipid mediators; while transcriptomics, single-cell RNA sequencing, and spatial transcriptomics can elucidate the functional and metabolic states of alveolar macrophages, neutrophils, T cells, and epithelial and endothelial cells (164–166), enabling the evaluation of candidate processes such as HIF-1α-mediated glycolysis, NLRP3 inflammasome activation, mtDNA/cGAS–STING signaling, and Treg/Th17 balance (167).

The value of multi-omics lies not merely in increasing measurement dimensions, but in testing whether reproducible source and functional connections exist between different observations. For candidate gut-to-lung effects, simultaneous assessment of intestinal microbiota or barrier changes, candidate signal generation, systemic effective exposure, target cell response, and pulmonary tissue or functional alterations should be pursued whenever possible. Strain-level source tracing, metabolite concentration gradients, and comparisons between portal/peripheral circulation and airway or pulmonary samples can help determine the source and direction of candidate signals. When conditions permit, stable isotope tracing, functional metabolomics, and perturbation experiments using patient-derived cells or organoids can further evaluate biological effects. Elevating cross-compartment correlations to functional cross-organ connections is scientifically justified only when the source, temporal sequence, effective exposure, and target tissue response can be aligned within a unified research framework. Conversely, if variations across different levels are primarily attributable to shared disease severity or treatment exposure, they should be interpreted as parallel host responses rather than evidence of a complete transmission mechanism.

### 10.3 Incorporating disease progression and ICU treatment exposure as time-varying factors in analysis

The microbiota, barrier, and immune–metabolic status of patients with severe pneumonia are continuously influenced by the disease course and ICU treatment. Future studies should systematically record key variables such as mechanical ventilation parameters, sedative and analgesic agents, neuromuscular blocking agents, antibiotic regimens (type and duration), enteral nutrition (initiation and volume), vasoactive support, and respiratory metrics including oxygenation status, hyperoxia exposure, and hypercapnia. These should be treated as time-varying covariates or effect modifiers rather than static baseline confounders.

Mechanical ventilation, in particular, should be decomposed into different physiological components, including tidal volume, plateau pressure, driving pressure, PEEP, mechanical power, FiO_2_, ventilation duration, patient–ventilator asynchrony, and weaning process. The direct pulmonary effects of mechanical stress on VILI should be modeled separately from the circulatory effects of positive pressure ventilation, as well as concomitant exposures such as sedation, nutrition, and antibiotics. Similarly, antibiotics may directly reshape the microbiota, nutrition affects microbial substrates and epithelial metabolism, while shock and vasoactive drugs alter splanchnic perfusion. Distinguishing these interrelated but mechanistically distinct exposures helps determine whether observed lung–gut connections arise from the common critical condition, treatment effects, or independent cross-organ associations. Meanwhile, host reserve, age, underlying diseases, pre-existing microbiome, infection burden, and organ function should be included in joint analyses to avoid misinterpreting the coexistence of more severe pulmonary, intestinal, and metabolic abnormalities in sicker patients as direct functional dependencies among these abnormalities.

### 10.4 Evaluating incremental clinical value and conducting mechanism-oriented interventional studies

Future research also needs to determine whether these cross-compartment indicators can provide additional information beyond current clinical assessments. Researchers should construct multi-system host response risk models that integrate intestinal barrier markers (e.g., D-lactate, DAO, I-FABP), microbiota characteristics of the gut and airway, metabolic indicators (including SCFAs, bile acids, tryptophan metabolites, and lactate), and inflammatory and treatment exposure variables. The focus should be on evaluating whether these indicators add predictive value for ARDS progression, VAP, prolonged mechanical ventilation, MODS, or mortality beyond traditional factors such as SOFA, APACHE II, shock, organ function, and mechanical ventilation parameters. Models should be assessed for discrimination, calibration, and clinical utility using internal and independent external validation cohorts. Stable predictive value can support risk stratification and study population enrichment but does not alone prove causal or interventional significance for the corresponding indicators.

On this basis, strategies such as early enteral nutrition optimization, microbiota interventions, lung-protective ventilation, anti-inflammatory or barrier-protective approaches should be evaluated separately according to mechanisms and patient phenotypes. Different interventions should be designed independently based on their respective biological targets and safety evidence, rather than being grouped as a unified “axis-targeted therapy.” In addition to clinical endpoints such as ARDS progression, VAP, duration of mechanical ventilation, MODS, mortality, and functional recovery, mechanism-oriented trials should include intermediate endpoints aligned with the hypothesized pathway. For example, if an intervention targets the intestinal barrier, further evaluation should assess whether barrier improvement is followed sequentially by changes in candidate circulating signals, target cell responses, and subsequent pulmonary functional changes. This joint evaluation of “local target change—systemic effective exposure—target tissue response—clinical outcome” helps distinguish mere biomarker changes from truly functional cross-organ effects. Ultimately, risk models, source tracing, functional experiments, and randomized interventional studies should be used together to determine which connections have only correlational or predictive value and which further possess independent biological and clinically actionable significance (**Table 3**).

**Table 3:** Future research priorities and clinical translation positioning of the gut-lung-immune-metabolic axis in severe Pneumonia.

Research priority direction	Core question	Recommended design and key elements	Current evidence maturity	Clinical translation positioning
Dynamic Longitudinal	Cohort Is microbial dysbiosis a disease driver, a concomitant change, or a treatment outcome?	Collect fecal, airway, and blood samples continuously at ICU admission, ventilation adjustment, infection control, and weaning stages, and synchronously record ARDS, VAP, MODS, and prognosis.	Low–Moderate: Evidence derived mainly from cross-sectional and indirect associations.	Clarify temporal sequence and screen candidate prognostic markers; currently does not support causal inference.
Source Tracing and Functional Validation	Do circulating microbial or metabolic signals originate from the gut and act on the lungs?	Conduct strain tracking, metabolite tracing, lung compartment detection, and blockade and rescue experiments in animals or organoids.	Exploratory/Preclinical: Lack of a complete continuous evidence chain.	To validate the “gut-derived signal–circulatory transport–lung injury” mechanism; no direct clinical application yet.
Multi-Omics Mechanistic Analysis	Which microbiota, metabolites, and immune cell subsets constitute key nodes?	Integrate metagenomics, metabolomics, single-cell, and spatial omics, focusing on SCFAs, bile acids, tryptophan metabolites, NLRP3, and cGAS–STING.	Exploratory: Candidate pathways exist, but cross-omics and independent validation are lacking.	Identify candidate biomarkers and intervention targets; not suitable for direct use as therapeutic basis.
ICU Treatment Exposure Adjustment	How to distinguish disease effects from changes caused by ventilation, antibiotics, sedation, and nutrition?	Include ventilation parameters, FiO_2_, antibiotics, sedation, nutrition, and vasoactive drugs as time-varying variables in causal models.	Methodological foundation required.	As a standard design element in observational studies and interventional trials to reduce confounding and misattribution.
Risk Stratification and Model Validation	Can relevant indicators improve risk prediction for ARDS, VAP, MODS, and mortality?	Add DAO, I-FABP, microbiota, metabolites, and inflammatory markers to SOFA or APACHE II, and conduct internal and external validation.	Early Clinical Research: No mature, generalizable model yet.	Used for high-risk identification and trial enrollment enrichment; cannot infer therapeutic benefit.
Biomarker-Stratified RCT	Can interventions targeting specific biological phenotypes improve real clinical outcomes?	Independently evaluate nutritional optimization, microbiome, or barrier protection strategies in addition to standard ICU care, while recording biomarkers and hard clinical endpoints.	Early Translation: Lack of sufficient severe pneumonia-specific RCTs.	Validate efficacy, safety, and applicable population; currently does not support routine targeted therapy.

“Low–Moderate” indicates some human association evidence exists, but longitudinal and causal validation is insufficient; “Exploratory/Preclinical” indicates evidence mainly from mechanistic studies or animal models; “Early Clinical Research/Early Translation” indicates a clinical research foundation exists but has not yet formed evidence for routine decision-making. Gut barrier, microbiome, and immune-metabolic indicators should currently be prioritized as candidate predictive or stratification variables, rather than presupposed pathogenic factors or therapeutic targets. APACHE II, Acute Physiology and Chronic Health Evaluation II; ARDS, Acute Respiratory Distress Syndrome; DAO, Diamine Oxidase; I-FABP, Intestinal Fatty Acid Binding Protein; MODS, Multiple Organ Dysfunction Syndrome; RCT, Randomized Controlled Trial; SCFAs, Short-Chain Fatty Acids; SOFA, Sequential Organ Failure Assessment; VAP, Ventilator-Associated Pneumonia.

## 11 Conclusion

Severe pneumonia-associated lung injury is not an isolated pulmonary event but occurs within a critically ill host environment shaped by infection burden, lung tissue damage, systemic inflammation, circulatory status, host immune metabolism, and multiple ICU treatment exposures. Current evidence indicates that dynamic changes occur in the lungs, intestines, circulation, and cellular immune metabolism during severe pneumonia and its treatment. However, whether these changes constitute a functionally interdependent cross-organ network must be assessed individually based on specific connections. The strength of evidence for different candidate connections is markedly asymmetric. There is sufficient physiological and clinical basis for the secondary effects of pulmonary infection, systemic critical illness, and hypoxia, hypoperfusion, and treatment exposure on the gut microbiome and barrier function. In contrast, consistent clinical evidence in humans that gut-derived microorganisms, metabolites, or inflammatory signals further result in significant pulmonary exposure and independently alter the progression of lung injury remains limited. The direct effect of mechanical ventilation in causing or exacerbating VILI is relatively clear, while its relationship with changes in the gut microbiome, barrier, and immune metabolism is influenced by multiple factors, including hemodynamics, sedation, antibiotics, nutrition, and disease severity. Therefore, the overall critical host response is more suitable as a foundational framework for explaining multisystem changes in severe pneumonia. The gut-lung-immune metabolism connection should be considered a candidate cross-organ relationship that requires further definition through temporal sequence, signal source and direction, effective exposure, target cell response, and functional dependence. Correlation or plausible mechanisms between different compartments is insufficient to establish functional integration. Its biological significance depends on whether the above evidence can form continuous support, and its clinical significance further depends on whether relevant indicators can provide stable incremental predictive value beyond disease severity, circulatory status, organ function, and treatment exposure, and whether interventions targeting specific mechanisms can produce reproducible biological and patient outcome changes. Lung-protective ventilation, infection control, sedation optimization, and rational nutrition should still be implemented based on their respective independent clinical evidence. Interventions related to the microbiome, barrier, and immune metabolism should be evaluated separately according to specific mechanisms and patient phenotypes. At this stage, the primary research value of the gut-lung-immune metabolism connection lies in proposing testable cross-organ hypotheses, identifying candidate risk markers, and clarifying possible functional connections, rather than serving as a unified disease mechanism or therapeutic target. Future research should further determine which cross-compartment associations can be validated as functional cross-organ connections with independent biological and clinical significance through longitudinal multi-compartment studies, source tracing, functional validation, and mechanism-oriented interventions.

## Glossary

AEC/AECs: alveolar epithelial cell(s)
AhR: aryl hydrocarbon receptor
ALI: acute lung injury
APACHE II: Acute Physiology and Chronic Health Evaluation II
ARDS: acute respiratory distress syndrome
ATP: adenosine triphosphate
CD14: cluster of differentiation 14
CECs: capillary endothelial cells
cGAMP: cyclic guanosine monophosphate–adenosine monophosphate
cGAS: cyclic GMP–AMP synthase
COVID-19: coronavirus disease 2019
CRP: C-reactive protein
DAMPs: damage-associated molecular patterns
DAO: diamine oxidase
DCs: dendritic cells
DNA: deoxyribonucleic acid
ECMO: extracorporeal membrane oxygenation
FFAR2: free fatty acid receptor 2
FFAR2/3: free fatty acid receptors 2 and 3
FFAR3: free fatty acid receptor 3
FiO₂: fraction of inspired oxygen
FMT: fecal microbiota transplantation
FXR: farnesoid X receptor
GI: gastrointestinal
GLUT: glucose transporter
GPR41: G protein-coupled receptor 41
GPR43: G protein-coupled receptor 43
GPR81: G protein-coupled receptor 81
GPR109A: G protein-coupled receptor 109A
HCAR1: hydroxycarboxylic acid receptor 1
HCAR2: hydroxycarboxylic acid receptor 2
HDAC/HDACs: histone deacetylase(s)
HIF-1α: hypoxia-inducible factor 1-alpha
HMGB1: high-mobility group box 1
HSPC: hematopoietic stem and progenitor cell
I-FABP: intestinal fatty acid-binding protein
IAP: intestinal alkaline phosphatase
ICU: intensive care unit
IFN-γ: interferon-gamma
IL-1β: interleukin-1 beta
IL-6: interleukin-6
IL-17A: interleukin-17A
IL-18: interleukin-18
IL-22: interleukin-22
IRF3: interferon regulatory factor 3
LBP: lipopolysaccharide-binding protein
LDHA: lactate dehydrogenase A
LPS: lipopolysaccharide
M2: M2 macrophage phenotype
Mo-Mac: monocyte-derived macrophage
MODS: multiple organ dysfunction syndrome
mtDNA: mitochondrial DNA
mtROS: mitochondrial reactive oxygen species
MV: mechanical ventilation
MyD88: myeloid differentiation primary response 88
NET/NETs: neutrophil extracellular trap(s)
NETosis: neutrophil extracellular trap formation
NF-κB: nuclear factor kappa B
NLRP3: NOD-like receptor family pyrin domain-containing 3
OXPHOS: oxidative phosphorylation
P.1: pathway 1
P.2: pathway 2
PAMPs: pathogen-associated molecular patterns
PaO₂: arterial partial pressure of oxygen
PaO₂/FiO₂: ratio of arterial partial pressure of oxygen to fraction of inspired oxygen
PEEP: positive end-expiratory pressure
pH: potential of hydrogen
Pplat: plateau pressure
PRRs: pattern-recognition receptors
RAGE: receptor for advanced glycation end products
RCT/RCTs: randomized controlled trial(s)
Rho-kinase: Rho-associated protein kinase
RNA: ribonucleic acid
ROS: reactive oxygen species
SARS-CoV-2: severe acute respiratory syndrome coronavirus 2
SCFA/SCFAs: short-chain fatty acid(s)
SOFA: Sequential Organ Failure Assessment
STING: stimulator of interferon genes
TBK1: TANK-binding kinase 1
TGR5: Takeda G protein-coupled receptor 5
Th17: T helper 17 cell
TLR: Toll-like receptor
TLR4: Toll-like receptor 4
TLR9: Toll-like receptor 9
TMAO: trimethylamine N-oxide
TNF-α: tumor necrosis factor-alpha
TRAM: tissue-resident alveolar macrophage
Treg: regulatory T cell
VAP: ventilator-associated pneumonia
VILI: ventilator-induced lung injury
VT: tidal volume
T17 cells: interleukin-17-producing gamma delta T cells
ΔP: driving pressure
ω-3: omega-3 fatty acids.
